# Comparative analysis of activation induced marker (AIM) assays for sensitive identification of antigen-specific CD4 T cells

**DOI:** 10.1371/journal.pone.0186998

**Published:** 2017-10-24

**Authors:** Samantha Reiss, Amy E. Baxter, Kimberly M. Cirelli, Jennifer M. Dan, Antigoni Morou, Audrey Daigneault, Nathalie Brassard, Guido Silvestri, Jean-Pierre Routy, Colin Havenar-Daughton, Shane Crotty, Daniel E. Kaufmann

**Affiliations:** 1 Division of Vaccine Discovery, La Jolla Institute for Allergy and Immunology, La Jolla, California, United States of America; 2 CR-CHUM, Université de Montréal, Montreal, Québec, Canada; 3 Center for HIV/AIDS Vaccine Immunology and Immunogen Discovery (CHAVI-ID), La Jolla, California, United States of America; 4 UCSD School of Medicine, Division of Infectious Diseases, La Jolla, California, United States of America; 5 Department of Pathology and Laboratory Medicine, Emory University School of Medicine, Yerkes National Primate Research Center and Emory Vaccine Center, Atlanta, Georgia, United States of America; 6 Chronic Viral Illnesses Service and Division of Hematology, McGill University Health Centre, Montreal, Québec, Canada; University of Hawaii System, UNITED STATES

## Abstract

The identification and study of antigen-specific CD4 T cells, both in peripheral blood and in tissues, is key for a broad range of immunological research, including vaccine responses and infectious diseases. Detection of these cells is hampered by both their rarity and their heterogeneity, in particular with regards to cytokine secretion profiles. These factors prevent the identification of the total pool of antigen-specific CD4 T cells by classical methods. We have developed assays for the highly sensitive detection of such cells by measuring the upregulation of surface activation induced markers (AIM). Here, we compare two such assays based on concurrent expression of CD69 plus CD40L (CD154) or expression of OX40 plus CD25, and we develop additional AIM assays based on OX40 plus PD-L1 or 4-1BB. We compare the relative sensitivity of these assays for detection of vaccine and natural infection-induced CD4 T cell responses and show that these assays identify distinct, but overlapping populations of antigen-specific CD4 T cells, a subpopulation of which can also be detected on the basis of cytokine synthesis. Bystander activation had minimal effect on AIM markers. However, some T regulatory cells upregulate CD25 upon antigen stimulation. We therefore validated AIM assays designed to exclude most T regulatory cells, for both human and non-human primate (NHP, *Macaca mulatta*) studies. Overall, through head-to-head comparisons and methodological improvements, we show that AIM assays represent a sensitive and valuable method for the detection of antigen-specific CD4 T cells.

## Introduction

Antigen-specific CD4 T cells play multiple key roles in immune responses to infection, vaccination, or self-antigen. Therefore, monitoring the development of such responses is crucial for many types of immunological research. The majority of conventional techniques detect antigen-specific CD4 T cells on the basis of cytokine production, either at the single cell level by flow cytometry or ELISpot, or at the population level by ELISA. However, these classical methods are limited by the cytokine detected, and are biased towards a particular type of CD4 T helper response. For example, standard panels are often Th1-skewed, predominantly staining for IFNγ and IL-2 [1,2]. CD4 T cell responses are remarkably heterogeneous, and thus detection of antigen-specific CD4 T cells by one or more cytokines produced is likely to significantly underestimate the size of the total antigen-specific response [3,4]. Furthermore, ICS and ELISpot assays only detect cells that produce cytokine above a certain threshold, although cells that produce cytokines below this threshold are likely to be biologically relevant.

Germinal center T follicular helper (GC Tfh) cells, a subset of CD4 T cells, are one such population that is largely missed or underestimated by classical cytokine-based detection methods [5,6]. Tfh cells are known to be critical for development of antibody responses [7,8], and thus detection of antigen-specific Tfh cells is critical for both understanding vaccine efficacy [9–11] and tracking disease progression [12]. Populations of circulating Tfh-like cells (cTfh) and memory Tfh cells have been identified in peripheral blood [12–16]. We, and others, have overcome the limitations of cytokine-based assays by designing CD4 T cell assays that define antigen-specificity on the basis of upregulation of TCR stimulation-induced surface markers rather than the production of cytokine, termed activation-induced marker (AIM) assays. We have developed an OX40/CD25 assay and have previously shown that this assay is highly efficient at detecting antigen-specific CD4 T cells [17,18], building upon earlier work that used whole-blood [19]. We have also developed a dual CD69/CD40L AIM assay [20], expanding on previous efforts utilizing CD40L alone [21,22]. Here, we present an in-depth comparison of those two AIM assays and show that both assays are able to detect distinct but overlapping populations of heterogeneous antigen-specific CD4 T cells. In addition, we address several potential concerns regarding the use of AIM markers for the identification of antigen-specific CD4 T cells, namely the possible contribution of bystander activation and the ability of such markers to detect CD4 T cells routinely identified by classical cytokine-based assays. Finally, we present new OX40/PD-L1 and OX40/4-1BB AIM assays for the detection of antigen-specific CD4 T cells in human and rhesus macaque samples. The comparative applications of these different AIM assays are discussed.

## Methods

### Human samples

Whole blood was obtained from study participants at the Montreal General Hospital and from healthy donors through the La Jolla Institute Normal Blood Donor Program (VD-057). LRS (leukoreduction) tubes were obtained through the San Diego Blood Bank. All participants provided informed written consent prior to enrollment in accordance with the respective Institutional Review Board approvals.

Subjects from the Montreal cohort were described as uninfected control donors with no HIV infection (UD), HIV-infected individuals receiving anti-retroviral therapy with a controlled viral load of <40 copies/ml (ART-T), HIV-infected participants who had not received treatment at the time of blood draw (UNT), and elite controllers with a controlled viral load of <40/copies/ml in the absence of anti-retroviral therapy (EC). All subjects from the Montreal cohort tested positive for human cytomegalovirus (hCMV) and were negative for Hepatitis C (HCV) and active Hepatitis B infection (HBV), although a subset had a resolved infection and/or received an immunization. PBMC were isolated by the Ficoll density gradient method and stored in liquid nitrogen.

### Non-human primate (NHP) ethics statement

All *Macaca mulatta* (rhesus macaque) study procedures were performed at Yerkes National Primate Research Center in strict accordance with the recommendations in the Guide for the Care and Use of Laboratory Animals of the National Institutes of Health. All protocols were approved by the Emory University Institutional Animal Care and Use Committee (IUCAC) (Permit No. A3180-01). All surgery was performed under anesthesia with ketamine or Telazol, and all efforts were made to minimize suffering [23]. There were no unexpected deaths in the course of this study.

### *M*. *mulatta* (rhesus macaque) lymphoid tissue and PBMC

Previously cryopreserved *M*. *mulatta* lymph node (LN) and PBMC were used. In short, *M*. *mulatta* were immunized subcutaneously with HIV BG505 SOSIP.v5.2 as previously described [18]. Lymph nodes were excised at necropsy, manually disrupted, and filtered with 70 μm strainers. Blood was also collected at necropsy in EDTA tubes and PBMC were isolated via gradient centrifugation.

### Antibodies

For details of antibodies used, see S1–10 Tables and descriptions of use below. In all cases, antibodies are monoclonal and raised in mice unless otherwise stated. All antibodies were validated and titrated using biological and/or isotype controls.

### OX40/CD25 assay

Cryopreserved human PBMCs from the Montreal HIV cohort were thawed, washed with AIM-V media (ThermoFisher Scientific), and rested at 37°C for 3 hours. Cells were aliquoted into wells of a 96-well plate, at a total of 1 x 10^6^ cells per well. For each assay, 5 conditions were used: no exogenous stimulation (“UN”), three antigen stimulations, or SEB (1 μg/ml). The antigen stimulations were overlapping peptide pools corresponding to one of HCMVA pp65 (human cytomegalovirus, JPT, PM-PP65), HIV-1 Gag ULTRA (JPT, PM-HIV-Gag), or HIV-ENV ULTRA (JPT, PM-HIV-ENV); the peptide pools were used to stimulate at a final concentration of 0.5μg/ml of total peptide. Following an 18 hr stimulation, the cells were stained for 1hr at 4°C according to the panel in S1 Table. The CXCR5 antibody was included during both the stimulation incubation and also included with the rest of the panel. The cells were washed, fixed with 1% formaldehyde, washed, and acquired the same day.

### CD69/CD40L assay

Matched samples from the Montreal cohort were thawed and processed as above in the OX40/CD25 assay, with the following modifications. The cells were rested overnight and a CD40 blocking antibody (Miltenyi Biotech, 130-094-133) was added to each group for 15 minutes prior to stimulation to a final concentration of 0.5 μg/ml. The cells were stimulated for 9 hours and stained with the panel in S2 Table.

### Combined OX40/CD25/PD-L1 and CD69/CD40L assay

Cryopreserved PBMCs were thawed, washed, resuspended at 10 x 10^6^/ml in RPMI + 10% Human AB Serum (HAB, Omega) and rested for 3 hours at 37°C. CD40 blocking antibody was added to a final concentration of 0.5μg/ml and cells were incubated for 15 min at 37°C. (Note: the use of a pre-conjugated anti-CD40L antibody, while a helpful alternative to the addition of CD40 blocking antibody, has the key caveat that the addition of monensin is required to prevent degradation of internalized antibody conjugates. This precludes the ability to use such an assay for live cell sorting and transcriptional analysis. We compared both approaches—anti-CD40L in culture vs. CD40 block—and achieved highly comparable results in terms of both frequency of antigen-specific cells and CD40L MFI). Antibodies against CXCR5 and CXCR3 were added directly to the culture. Cells were either left unstimulated (“UN”); stimulated with overlapping peptide pools corresponding to one of hCMVA pp65 (JPT, PM-PP65), HIV-1 Gag ULTRA (JPT, PM-HIV-Gag), HIV-ENV ULTRA (JPT, PM-HIV-ENV) or HBV large envelope (Hepatitis B Virus, JPT, PM-HBV-IEP); or stimulated with SEB as a positive control. All peptide antigens were used at a final concentration of 0.5 μg/ml. Cells were stimulated for 9 or 18 hours, collected and stained for 50 min at 4°C with the antibodies in S3 Table. Cells were washed, fixed for 20 min in 2% paraformaldehyde, washed, and resuspended in 1% FCS/PBS for flow acquisition.

### ICS + OX40/CD25 combined assay

*M*. *mulatta* lymph node cells were thawed and washed as described above in the OX40/CD25 Assay section and treated with DNase I (100 μg/ml, Stem Cell Technologies, 07900) for 15 minutes at 37°C prior to a 3 hour rest. Cells were split into three groups of 1 x 10^6^ each: no exogenous stimulation (“UN”), antigen stimulation (5 μg/ml BG505 Env protein and 5 μg/ml of each 15mer peptide of a peptide pool spanning BG505 Env), or SEB (1 μg/ml). The cells were incubated for 17 hours at 37°C, at which point Brefeldin A (Sigma) was added at a final concentration of 2 μg/ml; the cells were then incubated an additional 4 hours at 37°C. The cells were stained with the surface markers in S4 Table, washed, fixed with 1% formaldehyde for 20 minutes, washed, permeabilized with 0.5% saponin (Sigma, S7900) buffer, and intracellularly stained with the starred intracellular markers in S4 Table. Cells were then washed with FACS buffer and acquired the same day.

### Endotoxin stimulation in the OX40/CD25 assay

The OX40/CD25 assay as described above was performed on *M*. *mulatta* lymphoid tissue, with the addition of LPS (InVIVOGen, tlrl-k1h-4) at 25, 250, and 2500 EU/ml. Unstimulated (“UN”) and 1 μg/ml SEB samples were included as negative and positive controls. After 18 hours of incubation, the cells were stained according to the OX40/CD25 assay panel discussed above (S1 Table), washed, fixed with 1% formaldehyde, washed again, and acquired the same day.

### Detection of bystander activation using a transwell plate

PBMCs from a patient exposed to a proprietary phage antigen were cultured in the top wells of a 96-well transwell plate (Corning, PSHT004R1). Cryopreserved PBMC from healthy, antigen-naïve donors were cultured in the bottom wells. A 0.4 μm-pore size permeable membrane insert was used to enable free exchange of soluble factors between the top and bottom wells, while retaining cells within their designated wells. The cells were then cultured with media only (unstimulated condition, “UN”) or stimulated with the phage antigen (“Ag”) at 37°C for 24 hours as per the OX40/CD25 human PBMC assay. The cells were then stained according to the panel in S5 Table, washed, fixed with 1% formaldehyde, washed again, and acquired the same day.

### Generation of antigen-specific CD4 T cell lines

To generate antigen-specific, primary CD4 T cell lines, cryopreserved PBMCs from HIV-uninfected or HIV-positive individuals were thawed. CD8 T cells were depleted using Dynabeads CD8 Positive Isolation kit (Catalogue number 11333D) according to manufacturer's instructions. The negative fraction (CD8-depleted PBMCs) was washed, resuspended at 4 x 10^6^/ml in X-VIVO 15 (Lonza, BE02-053Q) + 10% HAB serum and rested at 37°C for 2 hours. Cells were stimulated overnight with the chosen peptide pool (either HIV-Gag peptide pool (JPT) or CMV pp65 (JPT); both 1 μg/peptide/ml). Following stimulation, the CD8-depleted PBMCs were washed, resuspended in fresh X-VIVO15 + 10% HAB + IL-2 (50 U/ml, NIH AIDS Reagent Program) at 2 x 10^6^/ml and plated in a 24 well plate (2 ml/ well). Cells were maintained for 10 days in culture, splitting when necessary, with IL-2 maintained at 50 U/ml. This long term culture and expansion was used to increase the frequency of antigen-specific CD4 T cells in the total CD8-depleted PBMC population. We therefore define these expanded cultures as CD4 T cell lines, although such cultures contain autologous APCs in addition to CD4 T cells. The cell line cells were then collected, washed, and resuspended in X-VIVO-15 + 10% HAB without IL-2. An aliquot of cells was reserved for antigen-specificity testing (described below), and the remaining cells were rested overnight before cryopreservation in liquid nitrogen.

### ICS for antigen-specificity testing of CD4 T cell lines

Antigen-specificity of the CD4 T cell lines generated above was determined using the “Combined OX40/CD25 and CD69/CD40L” approach described above and by ICS. For the ICS, briefly, 24 hr after IL-2 removal from the media (~Day 11 of culture) CD4 T cell line cells were collected, washed, and resuspended in X-VIVO + 10% HAB. The cell line was then restimulated with the appropriate antigen (0.5 μg/peptide/ml) for 6 hr. BFA/monensin was added after 1 hr. Cells were surface stained according to the panel in S6 Table, then fixed and permeabilized according to manufacturers instructions using eBioscience Intracellular Fixation and Permeabilization Buffer set (eBioscience, 88-8824-00). Cells were then stained intracellularly according to the panel in S6 Table, and resuspended in 1% FBS/PBS for acquisition.

### Detection of bystander activation using antigen-specific CD4 T cell lines

Aliquots of cryopreserved antigen-specific CD4 T cell line were thawed, washed in X-VIVO + 10% HAB and rested for 9–12hr at 37°C. The following day, autologous PBMCs were thawed and stained with a cell tracer (CellVue, eBioscience, 88-0875-17) according to manufacturer’s instructions, before resting for 3 hours at 37°C. Both the CD4 T cell line and labeled (CellVue^+^) autologous PBMCs were collected, counted and resuspended in X-VIVO + 10% HAB. The two cell types were plated onto 24 well plates either alone, or in the following ratios (Cell Line: Autologous PBMCs): 1:100, 10:100, 30:100, 100:100. In all cases the total number of cells per well was maintained at 5–10 x 10^6^/well in 1ml. CD40 blocking antibody (Miltenyi Biotech, as above) was added to a final concentration of 0.5 μg/ml and cells were incubated for 15 min at 37°C. In some experiments, antibodies against CXCR5 and CXCR3 were added directly to the culture (S3 Table). Cells were either left unstimulated, “UN”, or stimulated with SEB (0.5 μg/ml), or stimulated with the same peptide pool used for antigen-specific cell line generation (either HIV-1 Gag or CMV pp65, 0.5μg/peptide/ml) and cultured for 9 or 18hr. Following culture, cells were collected, stained, and processed as above (as in “Combined OX40/CD25 and CD69/CD40L assay” or “Treg identification in combined OX40/CD25 and CD69/CD40L assay”) using the panels indicated in S3 and S7 Tables, respectively.

### Treg cell identification in human PBMC

Cryopreserved human PBMCs from the San Diego Blood Bank were thawed, washed with RPMI + 5% HAB, and rested at 37°C for 3 hrs. Cells were then split into three groups of 1 x 10^6^ cells each: no exogenous stimulation (“UN”), tetanus peptide pool stimulation (1 μg/ml, see [24]), or SEB (100 ng/ml) and incubated for 18 hrs. Following stimulation, the cells were stained with the surface markers in S8 Table. The cells were then fixed with Fix/Perm Buffer (eBioscience, 00-5523-00), then stained for Foxp3 and Helios in Perm Buffer.

### Treg cell identification in combined OX40/CD25/PD-L1 and CD69/CD40L assay

In indicated experiments, markers for Treg cells were included in the “Combined OX40/CD25/PD-L1 and CD69/CD40L assay”. In these experiments, PBMCs from HIV-infected individuals were stimulated with various HIV peptide pools (HIV Gag Ultra (as above), HIV Nef Ultra (PM-HIV-NEF) or HIV Pol Ultra (PM-HIV-POL), all JPT) and surface stained using the panel described in S9 Table and protocol above. Cells were then fixed and permeabilized using eBioscience Fix/Perm Buffer (as above), according to manufacturers instructions, and stained intranuclearly with Foxp3 and Helios (see S9 Table) for 30 min at room temperature (RT). Cells were resuspended in 1% FBS/PBS for acquisition.

### Treg cell identification in *M*. *mulatta* PBMC and lymph node

The OX40/CD25 Assay as described above was performed on *M*. *mulatta* PBMC and lymph nodes. Cells were split into three groups of 1 x 10^6^ each: no exogenous stimulation (“UN”), antigen stimulation (5 μg/ml BG505 Env protein and 5 μg/ml of each 15mer peptide of a peptide pool spanning BG505 Env), or SEB (100 pg/ml, note that this is 1000x less than the concentration used for human PBMC samples, as recommended by [25]) and incubated for 18 hrs. The cells were then stained according to the panels in S10 or S11 Tables, fixed with Fix/Perm Buffer (eBioscience, above), and stained with Foxp3 and Helios in Perm Buffer.

### Flow cytometry

Cells were acquired on a BD Fortessa or LSRII Analyzer using FACSDiva^™^. Analysis was performed using FlowJo (TreeStar, Versions 9.9.4 and 10 for Mac).

### Calculations

In addition to considering the raw percentage of AIM^+^ cells within a sample, the frequency of antigen-specific cells is also presented variously as “background subtracted” or as “fold increase”. Background subtracted signal was calculated as the frequency of AIM^+^ cells in the antigen stimulation minus the frequency in the unstimulated condition. Fold increase was calculated as the frequency of AIM^+^ cells in the antigen stimulation divided by the frequency in the unstimulated condition.

### Statistical analysis

Statistical analyses were performed using GraphPad Prism 6 or 7. Data were tested for normal distribution and non-parametric tests were used in all cases. The Wilcoxon-Mann-Whitney test was used to compare groups. For correlation analysis, Spearman’s Rank Correlations were used.

## Results

### CD69/CD40L and OX40/CD25 AIM assays have comparable signal detection capabilities

**Fig 1:**
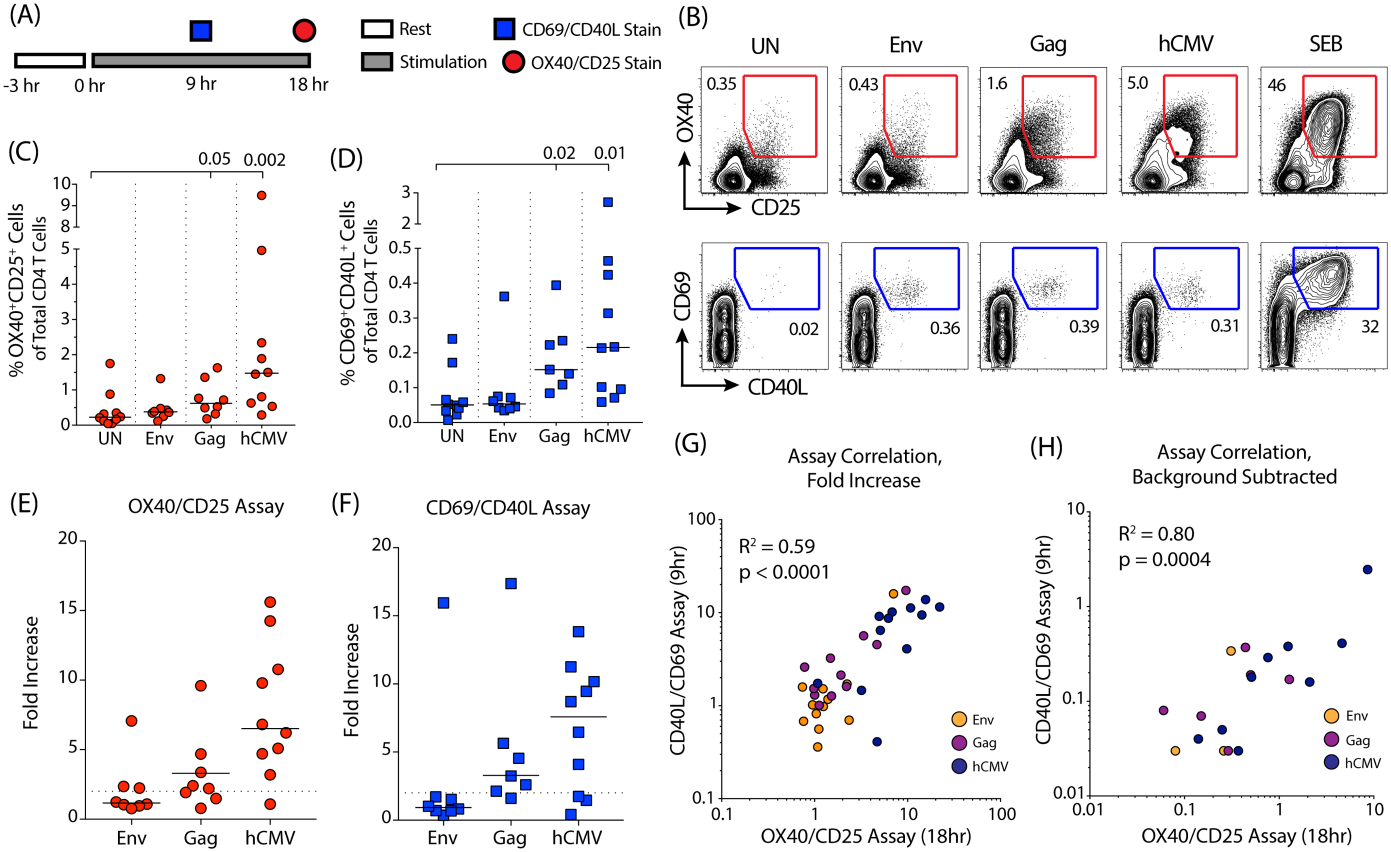
Comparing the OX40/CD25 and CD40L/CD69 AIM assays at their optimal time points. (A) A timeline of the assays’ setup and stimulation periods. (B) Example plots of OX40^+^CD25^+^ (red) and CD40L^+^CD69^+^ (blue) expression from human CD4 T cells when PBMC samples were unstimulated (UN), stimulated with peptides from HIV-Env, HIV-Gag, or hCMV, or stimulated with SEB as a positive control. Stimulation period was 9 hrs for the CD40L/CD69 assay and 18 hrs for the OX40/CD25 assay. Flow cytometry was gated as per S1 Fig (C,D) Quantitation of signal response to each antigen after 9 hrs or 18 hrs of stimulation for the CD40L/CD69 and OX40/CD25 assays, respectively. n = 8 for Env and Gag groups, n = 10 for NS and hCMV groups. *P* values shown comparing signal in the Gag and hCMV stimulations to the unstimulated (UN) condition for both assays (Wilcoxon test). (E,F) Quantitation of response measured by fold-increase (antigen-stimulation signal divided by the signal in the unstimulated control) for each antigen after 9 or 18 hrs of stimulation for the CD40L/CD69 and OX40/CD25 assays, respectively. Dotted line is shown at a fold-increase of 2, indicating positive signal threshold. (G,H) Correlation between the signals detected by the two assays after computing the fold increase and background subtracted values. The different antigen stimulations are shown in orange (Env), purple (Gag), or dark blue (hCMV). R^s^ = Spearman's Rank correlation coefficient with associated *p* values. For (E-H) n = 8 for Env and Gag groups, n = 10 for hCMV.

The OX40/CD25 and CD69/CD40L assays are independently developed tools designed to identify antigen-specific CD4 T cells via the detection of upregulated surface markers following antigen stimulation. To compare the two assays, human PBMCs from HIV-infected, CMV-positive individuals were stimulated and analyzed at the previously established stimulation times for each assay (Fig 1A; S1A and S1B Fig). HIV Env, HIV Gag, and hCMV stimulations were used to test a range of responses. The superantigen SEB was used as a positive control. Positive responses were defined here as greater than 2-fold over background in the “UN” condition [26] (defined as “fold increase” and calculated as the signal in the antigen stimulated condition divided by the signal in the unstimulated condition). Using these criteria, positive hCMV-specific CD4 T cell responses were detected by both assays (OX40/CD25 assay: 9 of 10 subjects; CD40L/CD69 assay: 7 of 10 subjects) with the median response being a 7-fold increase over background (Fig 1B–1F). The majority of subjects also had detectable Gag-specific responses: 5 of 8 donors in the OX40/CD25 assay and 6 of 7 donors in the CD69/CD40L assay, with a median 3-fold increase over background for both assays (Fig 1B–1F). Env-specific responses were rare but detectable, identified in 3 out of 8 donors in the OX40/CD25 assay and 1 of 8 donors in the CD69/CD40L assay (Fig 1B–1F). Overall, the fold increases in the size of the detected populations were similar between assays (Fig 1E and 1F).

While the signal in the antigen-stimulated conditions was higher in the OX40/CD25 assay, the level of background in the unstimulated condition was also higher (Fig 1C and 1D). Therefore, we calculated the background-subtracted signal (signal in the antigen-stimulation minus the signal in the unstimulated condition), in addition to the fold increase in signal, to assess the sensitivity and range of the two assays. The two assays had a high degree of correlation using both of these metrics (Fig 1G and 1H). When comparing the background subtracted signals between the two assays (Fig 1H), the OX40/CD25 assay identified antigen-responsive CD4 T cells in several donors/conditions for which a robust response was not detected by the CD69/CD40L assay. Both assays were successful at detecting antigen-specific CD4 T cell responses over a variety of antigens with good sensitivity and high concordance.

### CD69/CD40L and OX40/CD25 AIM assays identify overlapping populations of antigen-specific CD4 T cells

**Fig 2:**
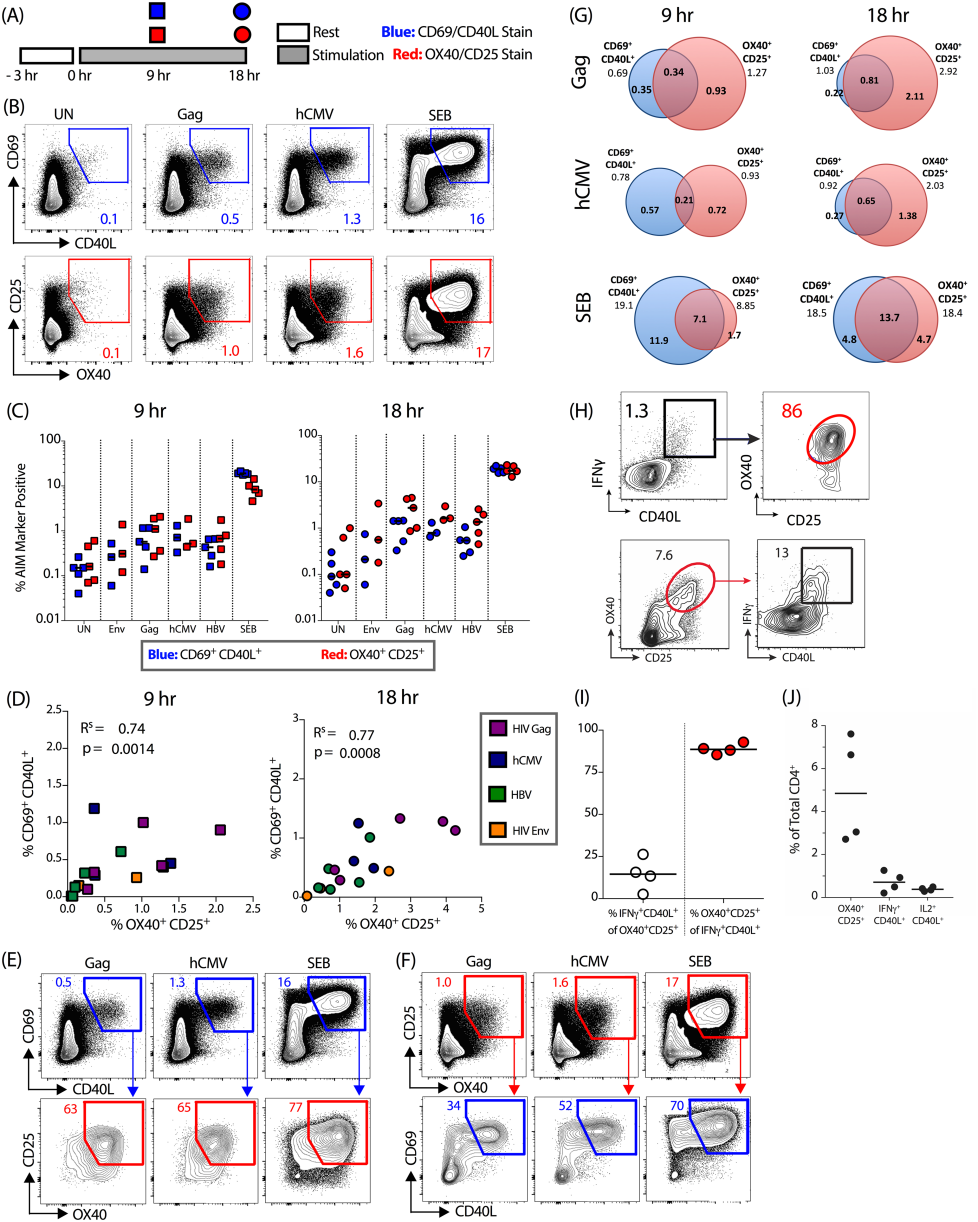
AIM markers identify overlapping populations of antigen-specific CD4 T cells. (A) Experiment protocol overview. Briefly, PBMCs were rested, then stimulated with antigen for 9-18hrs and stained for CD69, CD40L, OX40 and CD25 surface upregulation. (B) Example flow cytometry plots following 18-hr stimulation. (C) Quantification of data in (B) following a 9 hr (left) and 18 hr (right) antigen stimulation. CD69^+^CD40L^+^ is shown in blue and OX40^+^CD25^+^ in red. (D) Correlations between the two AIM assays following a 9 hr (left) and 18 hr (right) antigen stimulation. Data shown are background subtracted. Different antigens are indicated by color: HIV-1 Gag (purple), HIV-1 Env (orange), hCMV (dark blue) and HBV (green). R^s^ = Spearman's Rank correlation co-efficient with associated *p* values. (E,F) Example flow cytometry plots demonstrating the co-expression of different AIM at 18 hr post-antigen stimulation for (E) OX40/CD25 expression on CD69^+^CD40L^+^ CD4 T cells and (F) CD69/CD40L expression on OX40^+^CD25^+^ CD4 T cells. Numbers shown in bottom row indicate percentage from parent gate. (G) Quantification of data in (E,F). Venn diagrams illustrate overlap between the two AIM marker^+^ populations. All numbers represent mean percentages of AIM marker^+^ cells from total CD4 T cells. For (B-G) n = 3–5 HIV-infected, hCMV-positive donors per antigen. (H,I) Example flow cytometry plot showing OX40/CD25 expression on cytokine-producing cells (ICS CD40L^+^IFNγ^+^, top panels), and cytokine production from OX40^+^CD25^+^ cells (bottom panels), (*M*. *mulatta* PBMC). (I) Quantification of (H). (J) Detection of antigen-specific CD4 T cells by AIM vs. cytokine assays (*M*. *mulatta* PBMC). Cells from vaccinated animals were stimulated with BG505 antigen for a total of 21 hr with Brefeldin A added in the final 4 hrs. For H-J, n = 4 animals.

Given the difference in the magnitude of the antigen-specific CD4 T cell population identified by the two assays at different time points (Fig 1C and 1D), we next sought to determine the co-expression patterns of these four activation induced markers over time. PBMCs from 5 HIV-infected, hCMV-positive individuals were stimulated with antigen for either 9 or 18 hrs following addition of a CD40 blocking antibody, and then surface stained for OX40, CD25, CD69 and CD40L (Fig 2A). Importantly, the addition of CD40 blocking antibody to the culture had a minimal effect on the expression of markers other than CD40L or the frequencies of antigen-specific cells detected (S2A and S2B Fig). Following 9 hrs of antigen stimulation, the frequencies of hCMV-specific and HIV Gag-specific CD4 T cells were comparable between the two assays (Fig 2B and 2C). At 18 hrs, the OX40/CD25 assay identified a higher frequency of antigen-specific cells (non-background subtracted Fig 2B and 2C; background subtracted S1C Fig; fold increase S1D Fig). Both assays identified a similar number of positive responders (defined as at least 2-fold over background); an hCMV-specific response was detected in 3 out of 3 donors; a HIV-1 Gag-specific response in 5 out of 5 donors, and a HIV-1 Env-specific response in 2 out of 3 donors. For HBV, the OX40/CD25 assay identified HBV-specific responses in 5 of 5 donors compared to 3 of 5 donors for CD69/CD40L assay. At both time points, the frequencies of antigen-specific cells robustly correlated between the two assays (18 hr time point: r = 0.77, p = 0.0008; Fig 2D).

We next investigated the overlap between the two subsets of antigen-specific cells at both time points (Fig 2E and 2F). At 9 hrs, the two sets of markers identified only partially overlapping positive populations after antigen stimulation. However by 18 hrs the majority of CD69^+^CD40L^+^ CD4 were also OX40^+^CD25^+^ (Fig 2E and 2F). As this may be the result of the relatively slow upregulation of OX40, we concentrated further analysis on the 18hr time point. Following 18 hrs of HIV Gag stimulation, the majority of CD69^+^CD40L^+^ CD4 T cells were also positive for OX40^+^CD25^+^ (mean[range] = 76[63–92]%, Fig 2E–2G). A substantial fraction of OX40^+^CD25^+^ CD4 T cells were not CD69/CD40L double positive (e.g. for HIV Gag the proportion of antigen-specific CD4 T cells that were OX40^+^CD25^+^ only at 18 hr was mean[range] = 71[63–79]%; Fig 2F). However, the majority of this population was CD69^+^ (55–70%, Fig 2F, S2C Fig), indicating that CD40L expression differentiates these subsets. A similar pattern was observed following hCMV stimulation. Therefore, the two assays identify overlapping populations of antigen-responsive CD4 T cells.

To understand the overlap between cells that are detected by the OX40/CD25 AIM assay and a traditional ICS assay, stimulated *M*. *mulatta* PBMCs were stained for AIM surface markers and intracellular CD40L, IFNγ, and IL-2 by a delayed ICS, whereby a Golgi inhibitor was added during the final 4 hrs of antigen stimulation, allowing AIM markers to be sufficiently expressed on the surface during the preceding 17 hrs of stimulation. The vast majority of CD40L^+^IFNγ^+^ cells were also OX40^+^CD25^+^ (median = 89%, Fig 2H and 2I), confirming that cytokine-producing cells upregulate AIM markers. Conversely, the OX40/CD25 AIM assay also identified many additional antigen-specific cells that did not produce IFNγ and CD40L (Fig 2H). Within the OX40^+^CD25^+^ CD4 T cell population, less than 25% (median 15%) were positive for cytokine production (Fig 2I). This is consistent with the observation that the OX40/CD25 assay identified between 3- and 6-fold more antigen-specific CD4 T cells than a classical IFNγ and CD40L ICS assay (Fig 2J). Thus, the OX40/CD25 AIM assay identifies an antigen-responsive CD4 T cell population that includes cells identified by conventional ICS, as well as additional antigen-responsive cells.

### Bystander activation has a limited effect on the upregulation of AIM markers on CD4 T cells

**Fig 3:**
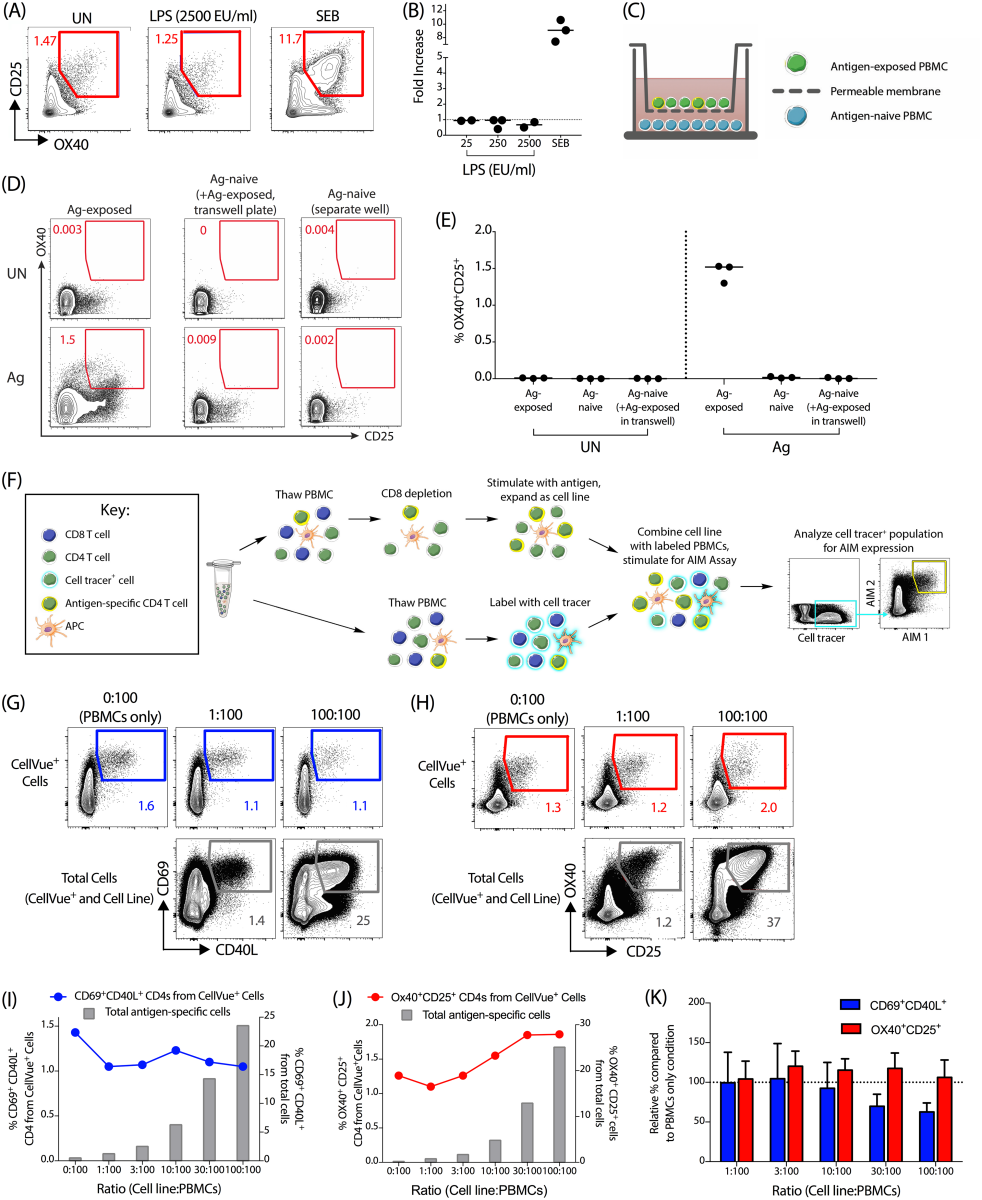
Bystander activation by contaminants or non-TCR stimulation has limited impact on upregulation of AIM markers. (A,B) OX40/CD25 expression of PBMCs cultured in the presence of increasing amounts of LPS (25–2500 EU/ml), with an unstimulated (UN) and SEB condition as negative and positive control. (A) Example plots from a representative donor cultured with or without LPS. (B) Data shown as fold-increase (LPS/UN); n = 3 donors. (C) Experimental setup for transwell plate experiment, where antigen-exposed PBMCs were placed within the top well of a transwell plate, and antigen-naïve PBMCs were placed within the bottom well, with the two wells separated by a 0.4 μm pore-size permeable membrane. The cultures were then left unstimulated (UN) or stimulated with antigen (Ag) for 24 hours at 37°C. (D) Representative flow cytometry plot of the antigen-experienced PBMCs in the top well of the transwell plate (left column), the antigen-naïve PBMC in the bottom well of the transwell plate (middle column), and the antigen-naïve PBMC control in a separate plate (right column) after 24 hours in culture; with the unstimulated condition (UN) on the top row and antigen stimulated (Ag) on the bottom row. Gated on total CD4 T cells. (E) Quantification of the OX40^+^CD25^+^ signal for the three culture conditions, unstimulated (UN) or stimulated with antigen (Ag). n = 3 antigen-naïve donors. (F) Diagram of the CD4 T cell line bystander activation experiment from panels G-K. Briefly, cell tracer-labeled (CellVue^+^) PBMCs were cocultured with an autologous CD4 T cell line at different ratios and stimulated with peptide antigens for 18 hr. (G,H) Example flow plots show the frequency of antigen-specific CD4 T cells CellVue^+^ PBMCs in the top row compared to the total frequency of antigen-specific cells within the coculture (CD4 T cell line + CellVue^+^ PBMCs) in the bottom row, at indicated ratios of CD4 T cell line and CellVue^+^ PBMCs. Antigen-specific cells are identified as CD69^+^CD40L^+^ (G) or OX40^+^CD25^+^ (H) respectively. (I,J) Quantification of data shown in (G,H) for CD69/CD40L (I) and OX40/CD25 expression (J) respectively. The total frequency of antigen-specific cells in the culture (CD4 T cell line + CellVue^+^ PBMCs) is indicated by grey bars; the frequency of CellVue^+^ autologous CD4 T cells from PBMCs is indicated by the colored dots/lines. (K) Data shown as a relative change in the percentage of antigen-specific CD4 T cells from the CellVue^+^ PBMCs at each ratio, compared to the PBMC alone condition. n = 3 independent experiments and donors, with HIV-Gag or hCMV pp65 peptide stimulation. Bars represent mean +/- SEM, dotted line at 100% (i.e. no change).

One potential concern with the use of AIM markers for identification of antigen-specific cells is that non-TCR mediated upregulation of surface markers could potentially be caused by cell culture contaminants or factors secreted by bystander cells and may lead to false-positive results. First, we investigated the effect of LPS on OX40 and CD25 upregulation on CD4 T cells over a range of concentrations. The highest LPS concentration tested (2500 EU/ml) was approximately 5-fold higher than the amount of endotoxin commonly found in synthetic peptide preparations used in T cell stimulation assays. No increase in the size of the antigen-specific population over the unstimulated condition was observed with any concentration of LPS (Fig 3A and 3B). In comparison, SEB stimulation gave a robust response (Fig 3A and 3B). Therefore, cell culture contamination by LPS is unlikely to cause bystander activation in the OX40/CD25 AIM assay.

To address the potential impact of bystander activation by cell-secreted factors, such as cytokines, we performed two complementary sets of experiments. Firstly, we utilized a transwell plate system to investigate the effect of co-culturing cells from an antigen-experienced donor with those from an antigen non-exposed donor (Fig 3C). Stimulation of antigen-specific CD4 T cells had no effect on the expression of OX40 and CD25 on the antigen-naïve CD4 T cells, compared to antigen-naïve CD4 T cells stimulated alone (Fig 3D and 3E). This indicated that OX40 and CD25 are not affected by soluble factors secreted by nearby antigen-specific cells at amounts typically experienced during the course of this assay.

While these results were promising, the requirement for a heterologous culture prevented the assessment of a potential role of cell-to-cell contact. Furthermore, if limited bystander activation was observed in the presence of an exceptionally high frequency of antigen-specific cells, that would provide an additional degree of confidence in the specificity of the assay. To investigate these points, we generated antigen-specific cell lines from CD8-depleted PBMCs against HIV-1 Gag or CMV pp65 peptide antigens (see Fig 3F). These primary cell lines contain very high frequencies of antigen-specific CD4 T cells (~30–60%, as measured by ICS for TNFα production, S3A Fig), in addition to autologous APCs, and therefore they are defined here as CD4 T cell lines. Each CD4 T cell line was subsequently mixed with autologous primary PBMCs labeled with a cell tracer at increasing ratios; these labeled (CellVue^+^) primary PBMCs represent the population where any bystander activation can be measured. The co-culture was then re-stimulated with the same antigen used to generate the CD4 T cell line (i.e. either HIV-1 Gag or CMV pp65) for 18 hrs and the combined OX40/CD25 and CD69/CD40L AIM assay performed. We first confirmed that there was limited toxicity associated with the co-culture of the cell line and primary PBMCs (S3B Fig). Both the total frequency of antigen-specific cells within the culture (representing the combined CD4 T cell line and primary PBMCs; grey bars) and the frequency of CellVue^+^ CD4 T cells (representing primary PBMCs only; colored dots and line) were determined (Fig 3G–3J). As shown for an example donor (Fig 3G and 3I), we observed no increase in the size of the CD69^+^CD40L^+^ primary CD4 population, even at the highest ratio of CD4 T cell line to PBMCs (100:100). In this example, at this ratio, >20% of the total cells in the culture are antigen-specific. We did observe a slight increase in the frequency of OX40^+^CD25^+^ CellVue^+^ CD4 T cells (Fig 3H and 3J), however, this occurred only above a 10:100 ratio of CD4 T cell line to PBMCs, corresponding to approximately 5% total antigen-specific cells in the co-culture (see grey bars, Fig 3J). Even when the total frequency of a antigen specific cells in the culture was ~15–40%–substantially above frequencies classically found in peripheral blood—we detected only a very minor increase in the frequency of OX40^+^CD25^+^ CellVue^+^ CD4 T cells (from 1.26% to 1.86%). This is particularly striking when taken in the context of a very high frequency of total antigen-specific cells in the culture. Across multiple experiments, the frequency of antigen-specific CellVue^+^ CD4 T cells remained relatively consistent following cell line addition when compared to the PBMC alone condition (Fig 3K). We therefore conclude that bystander activation, mediated either through soluble factors or cell-to-cell contact, has a limited effect on the detection of antigen-specific CD4 T cells by either set of AIM markers.

### Regulatory T cells in human and non-human primate (NHP) AIM assays

**Fig 4:**
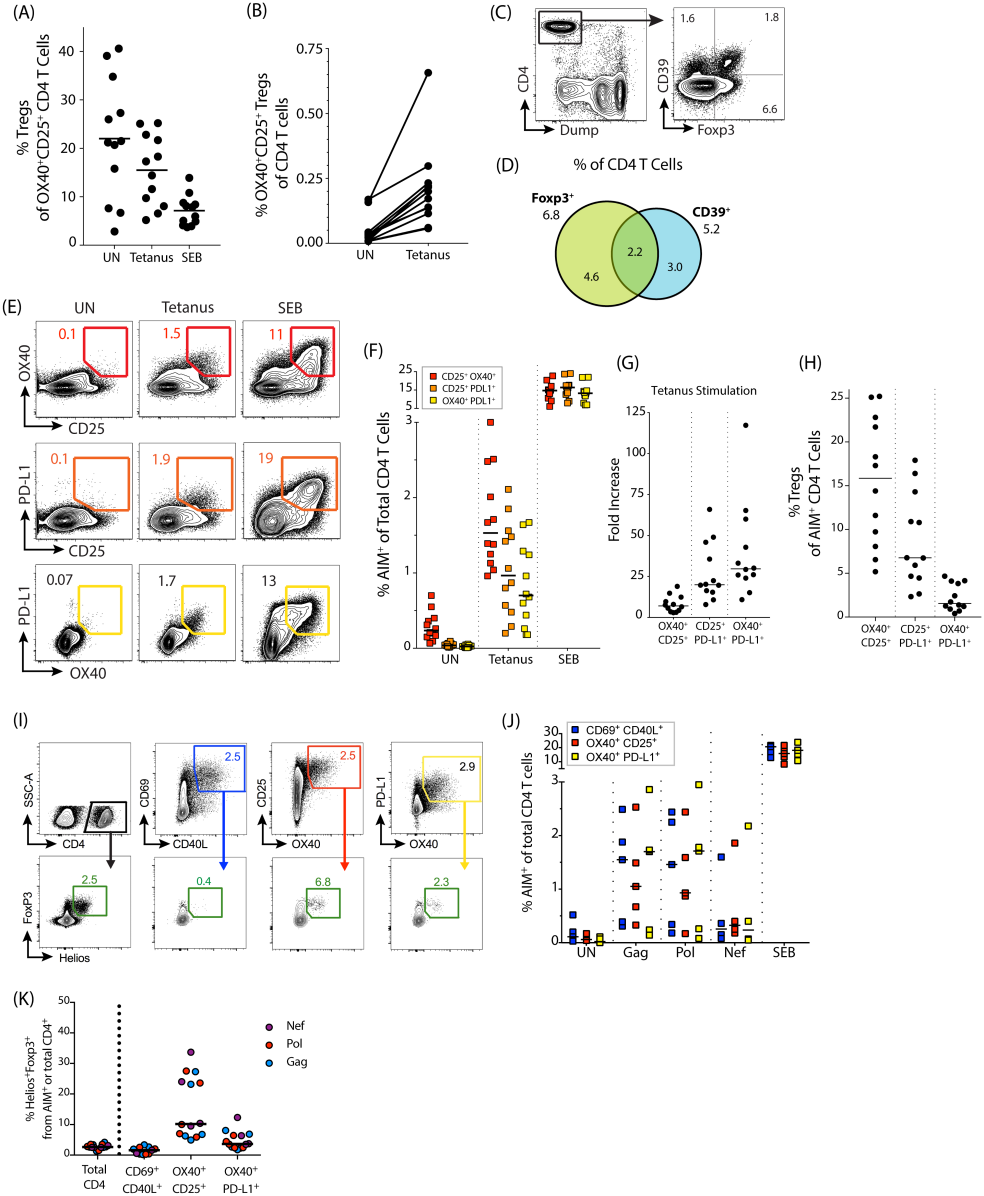
Treg cells in human PBMC. (A) Graph showing the frequency of Foxp3^+^ Treg cells within the AIM^+^ total CD4 population after 18 hrs of no stimulation (UN) or stimulation with tetanus peptide pool. (B) Increasing amounts of OX40^+^CD25^+^ nTreg cells (Foxp3^+^Helios^+^) within the total CD4 T cell population in human PBMC in response to tetanus peptide pool stimulation. (C) Example plot of CD39 and Foxp3 expression of total CD4 T cells in an unstimulated human PBMC sample after 18 hrs of incubation at 37°C. (D) Venn diagram showing overlap between Foxp3 and CD39 expression on total CD4 T cells in unstimulated human PBMC (18 hrs at 37°C). Numbers shown are mean percentages; n = 12. (E) Example plots of signal detected using different combinations of AIM markers: OX40/CD25 in red, PD-L1/CD25 in orange, and PD-L1/OX40 in yellow. (F) Quantitation of AIM response via different marker combinations after stimulation with tetanus peptide pool. (G) Fold increase in signal for the different marker combinations (tetanus peptide pool stimulation response divided by the unstimulated condition). (H) Graph showing the percent of Foxp3^+^ Treg cells within the AIM^+^ total CD4 population in tetanus stimulated human PBMC where the AIM^+^ population is defined by different combinations of markers. For A, B, E, F, G, and H, n = 12 donors. (I-K) PBMCs from 5 HIV-infected donors stimulated for 18 hrs with various HIV peptide antigens (Gag, Pol, and Nef). Antigen-specific CD4 T cells were identified by the upregulation of AIM markers and analyzed for the contributions of Helios^+^Foxp3^+^ Tregs. n = 4–5 donors. (I) Example flow plots showing gating of AIM^+^ populations, and subsequent identification of AIM^+^ Foxp3^+^Helios^+^ Tregs, following Gag stimulation (all plots from same donor). (J) Quantification of AIM^+^ signal from the various marker combinations shown in the top panel of (I). (K) Quantification of Helios^+^Foxp3^+^ Tregs within AIM^+^ gates. Treg data shown only where antigen-specific responses were 2-fold over background (2–5 responses per antigen).

Treg cells may contribute to the frequency of AIM maker positive (AIM^+^) cells detected with the OX40/CD25 assay. While approximately 22% of the small number of OX40^+^CD25^+^ CD4 T cells in the unstimulated condition of human PBMC are Foxp3^+^ (Fig 4A), this fraction decreases upon TCR stimulation, indicating that the majority of the AIM^+^ signal comes from antigen-specific non-Treg cells (Fig 4A). However, a population of antigen-responsive Treg cells is still detected, as evidenced by quantifying OX40^+^CD25^+^ Foxp3^+^Helios^+^ Treg cells in response to tetanus peptide pool stimulation (Fig 4B).

To further refine the OX40/CD25 assay to specifically distinguish Treg cells without requiring an intranuclear stain (e.g. Foxp3 or Helios) or a CD40 block, we explored the use of additional surface markers. CD39 has been shown to enrich for Foxp3^+^ Tregs within OX40^+^CD25^+^ antigen-specific CD4 T cells [19]. While we observed moderate expression of CD39 on Treg cells, roughly half of CD39^+^ CD4 T cells were negative for Foxp3, and the majority of Treg cells did not express CD39 (Fig 4C and 4D). We therefore concluded that CD39 was not sufficient to identify Treg cells in conjunction with the OX40/CD25 AIM assay with sufficient specificity.

We next investigated PD-L1 as a potential AIM marker to discriminate antigen-specific non-Treg cells. PD-L1 is an activation-induced cell marker in humans and has been successfully used in conjunction with OX40 or CD25 to identify antigen-specific cells (Fig 4E, see [18]). When the co-upregulation of combinations of OX40, CD25, and PD-L1 were compared after tetanus peptide stimulation, we found that OX40 and CD25 expression detected the largest population of activated cells. While OX40 combined with PD-L1 detected fewer total antigen-specific cells upon stimulation, the frequency of positive cells in the unstimulated condition was also markedly lower (Fig 4E and 4F). Consequently, the use of OX40 combined with PD-L1 resulted in the highest fold increase over the unstimulated condition in the detection of antigen-specific cells, followed by CD25 plus PD-L1, and then OX40 plus CD25 (Fig 4G). More importantly, very few Foxp3^+^ Treg cells were detected in the antigen-responsive OX40^+^PD-L1^+^ gate following tetanus peptide pool stimulation, compared to the OX40^+^CD25^+^ or CD25^+^PD-L1^+^ gates (median 1.6%, compared to 6.8% and 16%, respectively; Fig 4H). Therefore, OX40 combined with PD-L1 improves the overall specificity of the OX40-based assays by primarily detecting non-Treg antigen-specific CD4 T cells.

Given this, we then compared both OX40-based AIM assays with the CD69/CD40L AIM assay in PBMC from HIV-infected donors (Fig 4I). We found that the OX40/PD-L1 and CD69/CD40L assays had comparable sensitivities and specificities at the 18 hr time point for a range of HIV antigens (Fig 4J). Both the CD69/CD40L and OX40/PD-L1 combinations had much lower levels of Foxp3^+^Helios^+^ Tregs within the AIM^+^ CD4 T cell populations compared to the OX40^+^CD25^+^ population, with the CD69/CD40L combination containing the fewest Tregs (Fig 4K). In conclusion, the OX40/CD25 marker combination identifies both non-Treg antigen-specific CD4 T cells and ‘antigen-responsive’ Treg cells, while the OX40/PD-L1 and CD69/CD40L combinations more discriminately detect antigen specific non-Treg CD4 T cells.

**Fig 5:**
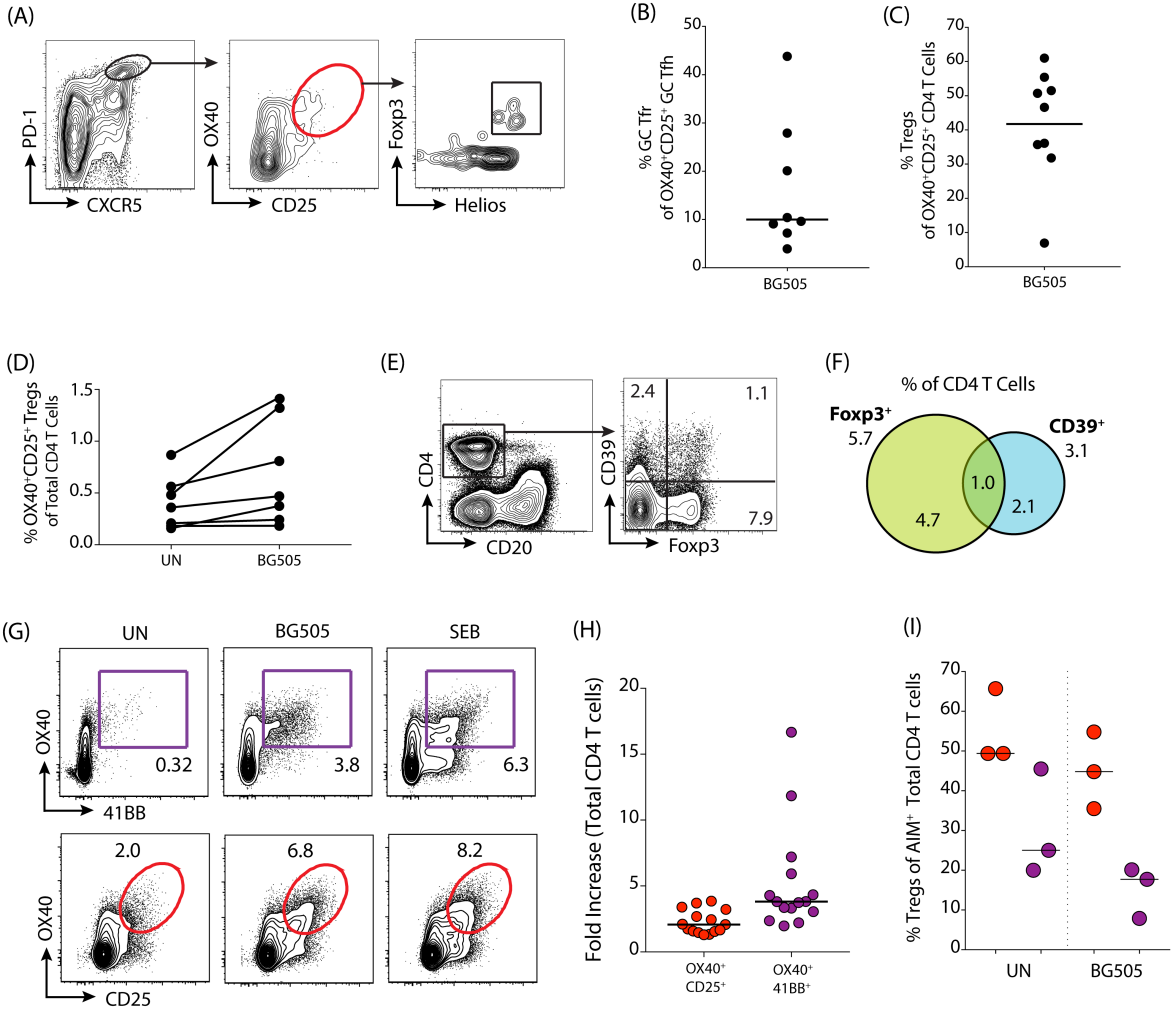
Treg cells in *M*. *mulatta* PBMC and lymph nodes. (A) Example gating scheme showing Foxp3 and Helios expression in OX40^+^CD25^+^ GC Tfh (PD-1^hi^CXCR5^hi^) from *M*. *mulatta* lymph node. (B) Quantitation of the percent of Treg cells within the OX40^+^CD25^+^ GC Tfh population after stimulation with antigen (BG505) (*M*. *mulatta* LN, n = 8 animals). (C) Quantitation of Treg cells within the OX40^+^CD25^+^ total CD4 T cell population after stimulation with antigen (BG505) (*M*. *mulatta* PBMC, n = 9 animals). (D) Frequencies of OX40^+^CD25^+^ nTreg cells (Foxp3^+^Helios^+^) within the total CD4 T cell population in *M*. *mulatta* PBMC compared between the unstimulated condition “UN” and in response to BG505. n = 7. (E) Example plot of CD39 and Foxp3 expression of unstimulated total CD4 T cells in *M*. *mulatta* PBMC after 18 hrs of incubation at 37°C. (F) Venn diagram demonstrating overlap between Foxp3 and CD39 expression on total CD4 T cells in *M*. *mulatta* PBMC after 18 hrs of incubation at 37°C without stimulation. Numbers shown are mean; n = 7. (G) Example gating scheme for detection of OX40 and 4-1BB (purple) or OX40 and CD25 (red) AIM^+^ CD4 T cells in *M*. *mulatta* LN. (H) Fold-increase (signal from the antigen condition divided by the unstimulated condition) for the different marker combinations: OX40^+^CD25^+^ (red) or OX40^+^4-1BB^+^ (purple) (*M*. *mulatta* PBMC and LN, n = 15). (I) Comparison of Treg cell frequencies within the AIM^+^ CD4 T cell population for the different marker combinations: OX40^+^CD25^+^ (red) or OX40^+^4-1BB^+^ (purple) (PBMC, incubated at 37°C for 18 hrs. n = 3).

Tfr cells are Treg cells with Tfh characteristics (i.e. CXCR5^+^Bcl6^+^ [27]). In *M*. *mulatta* lymph node (LN) cells, we confirmed that among antigen-specific GC Tfh cells (as identified by PD-1^hi^CXCR5^hi^ OX40^+^CD25^+^; Fig 5A) GC Tfr cells (additionally defined as Foxp3^+^Helios^+^ within this population) are relatively infrequent (median[range] = 10[4–44]% of BG505-stimulated GC Tfh cells, Fig 5B), reinforcing the utility of the OX40/CD25 AIM assay for identification of antigen-specific GC Tfh cells [17,18]. However, within the total CD4 T cell population in *M*. *mulatta* PBMCs and LNs, approximately half of the responding OX40^+^CD25^+^ cells were Foxp3^+^Helios^+^ (40–60% in PBMC and LN; Fig 5C, S4A and S4B Fig). The OX40/CD25 assay identifies antigen-responsive Treg cells in addition to antigen-specific CD4 T cells in *M*. *mulatta* PBMC (Fig 5D) and LN (S4C Fig). In conclusion, the OX40/CD25 AIM assay identifies antigen-specific GC Tfh cells effectively, but a substantial frequency of Treg cells are detected when total CD4 T cells are analyzed.

We therefore examined additional surface markers that could enable us to distinguish Treg cells when studying *M*. *mulatta* CD4 T cell responses in parallel to our human CD4 T cell studies described above (Fig 4). In *M*. *mulatta* PBMC, CD39 exhibited very little overlap with Foxp3 in CD4 T cells (Fig 5E and 5F). Results were similar in *M*. *mulatta* LN (S4D and S4E Fig). Therefore, we explored additional AIM markers to specifically identify the non-Treg population of antigen-specific CD4 T cells. As described above, the OX40/PD-L1 AIM assay specifically identifies antigen-specific CD4 T cells. However, commercially available anti-human PD-L1 antibodies are minimally cross-reactive to *M*. *mulatta* PD-L1. Therefore, we tested 4-1BB, another marker originally identified in the *M*. *mulatta* GC Tfh RNAseq screen [18]. After stimulation with antigen or SEB, 4-1BB was strongly upregulated in combination with OX40 on CD4 T cells (Fig 5G). Very few cells were 4-1BB^+^ in the unstimulated condition, resulting in a higher fold-increase using OX40^+^4-1BB^+^ gated cells than OX40^+^CD25^+^ cells (Fig 5H). In addition, the OX40^+^ 4-1BB^+^ CD4 T cell population contained substantially fewer Treg cells than the OX40^+^CD25^+^ CD4 T cell population in both the unstimulated condition and the antigen-stimulated condition (Fig 5I). We conclude that the combination of 4-1BB and OX40 is the most sensitive marker combination for the AIM assay in *M*. *mulatta*, and specifically identifies non-regulatory antigen-specific CD4 T cells when compared to OX40/CD25. 4-1BB was tested for human T cell AIM, and contrary to our results in *M*. *mulatta*, had higher levels of background in the unstimulated condition than the OX40/PD-L1 combination (S4F and S4G Fig). Thus we conclude that there are different optimal AIM markers for antigen-specific human and *M*. *mulatta* CD4 T cells.

## Discussion

The detection of antigen-specific CD4 T cells represents a key challenge in detection of vaccine or infection-induced responses. Detection of an antigen-specific CD4 T cells is also a major challenge in the context of autoimmune diseases. CD4 T cell responses are remarkably heterogeneous and only a subset of antigen-specific cells can be detected by measuring cytokine production. We, and others, have developed surface marker based AIM assays to identify rare antigen-specific CD4 T cells in a robust and sensitive manner that is cytokine independent. Here, we cross-compared multiple such assays in two laboratories, addressed bystander activation concerns, and further optimized the AIM assays. The data show that AIM assays are robust; we observed a high degree of agreement between the CD69/CD40L and the OX40/CD25 assays with regards to the magnitude of antigen-specific CD4 T cell responses and relative donor response rates at both research sites. The AIM^+^ populations identified in each of these assays were largely overlapping, but not identical, and contained the vast majority of cytokine-producing cells. The presence of high frequencies of activated CD4 T cells, or the presence of LPS, had minimal impact on the antigen-specific signal. Thus, AIM assays are robust, specific assays for measuring antigen-specific CD4 T cells.

When compared to standard ICS assays, the identification of antigen-specific cells by AIM assays has multiple advantageous features for addressing specific experimental questions. Firstly, antigen-specific CD4 T cells are heterogeneous and not all produce the classical Th1/2/17 cytokines detected in conventional assays. Therefore, ICS and ELISpot assays underestimate the size of the antigen-specific response and miss subpopulations of antigen-specific CD4 T cells. In particular, Tfh cells produce lower amounts of cytokines than their Th1/2/17 counterparts, and as such are substantially under-detected in cytokine assays [17,18]. As AIM assays are based on TCR-dependent activation marker upregulation, they capture a non-polarized, cytokine-independent heterogeneous response, including cells such as Tfh cells and weakly polarized cells, such as those generated by pertussis [17] or tetanus immunization [24]. An important additional feature of AIM assays is that the antigen-specific cells are alive at the end of the assay. These cells can thus be used for multiple downstream applications, such as gene expression analysis by RNAseq after live sorting [11], TCR identification, functional assays, and T cell cloning. AIM assays therefore increase the amount of information that can be obtained from rare cells. This can be of particular importance in clinical trial settings.

AIM assays are highly flexible; both the AIM markers used and the time point of analysis can be altered depending on the experimental questions to be addressed and researcher priorities. For gene expression experiments, the time point at which responses are analyzed post-antigen stimulation can be key. Due the differential kinetics of the various AIM markers, the CD69/CD40L assay is particularly valuable at earlier time points (6–9 hrs). In contrast, robust upregulation of OX40 requires at least 12 hours. Analysis at 6–9 hrs may be best for the identification of early upregulated genes, such as the first wave of a cytokine response. Alternatively, the CD69/CD40L, OX40/PD-L1, and OX40/CD25 assays can all be used following a longer stimulation (18–24 hrs).

We recommend the OX40/PD-L1 assay as a convenient two-marker assay that sensitively identifies antigen-specific CD4 T cells in both peripheral blood and LNs, including GC Tfh cells, which are notoriously difficult to identify. The CD69/CD40L version is another excellent option as a two-marker assay that robustly identifies antigen-specific CD4 T cells in peripheral blood at comparable frequencies and sensitivities to the OX40/PD-L1 assay. Additionally, the CD69/CD40L AIM assay detects an important functional helper molecule of CD4 T cells, CD40L. If the primary experiment endpoint is to determine the maximal size of the total antigen-responsive population, we recommend the use of the OX40/CD25 assay. However, this population will contain some amount of Treg cells. Thus, a combination of OX40/PD-L1/CD25 or CD69/CD40L can be used to identify the non-Treg and Treg AIM^+^ populations. For absolute exclusion of antigen-responsive Treg cells from any of the AIM^+^ CD4 T cell populations, staining for the canonical Treg protein Foxp3 provides the most robust identification of this subset. However, intra-nuclear staining then precludes the use of the sample for additional downstream applications. Therefore, the choice of marker combination(s) depends on whether it is more important to maximize the size of the detected response, to focus on the CD4 T cell subset with likely effector function, or to identify the Treg subset within the antigen-responsive population—and if the latter, how stringently the subsets must be distinguished. Furthermore, other groups have recently built on the AIM assay platform using CD69 and CD200 to identify antigen-specific cells within a vaccination-induced CD38^+^ICOS^+^ cTfh population [28], presenting a further alternative to those discussed here.

The majority of the work discussed here focuses on identification of antigen-specific CD4 T cells in PBMC. However it is increasingly well accepted that measurements in the peripheral blood do not provide a complete picture of the ongoing immunological responses in the tissues [29]. To this end, fine needle aspirates (FNAs) and surgical biopsies are increasingly being used to enable the study of LN cells and other lymphoid tissues in both humans and NHPs [11,30–33]. The study of tissue samples rather than peripheral blood affects AIM assay marker choice. In LNs, CD69 is constitutively expressed on GC Tfh cells, and therefore cannot be used as an AIM marker for this subset [17,18]. Instead, we recommend the OX40/PD-L1/CD25 assay for human lymphoid tissue studies and the OX40/4-1BB/CD25 assay for *M*. *mulatta* lymphoid tissue studies.

Overall, AIM assays are flexible, live-cell, easy to employ assays capable of discriminating CD4 T effector versus regulatory responses, which enable the study of a broad range of antigen-specific CD4 T cells that have previously gone undetected.

## Supporting information

S1 Fig Comparison of AIM assays at multiple time points. (A) Example flow plot gating strategy for the CD40L/CD69 Assay (dump channel contains a viability dye, CD8, CD14, CD16, and CD20) shown for HIV-Gag stimulated PBMCs. (B) Example flow plot gating strategy of the OX40/CD25 Assay shown for HIV-Gag stimulated PBMCs. (C,D) Quantification of antigen-specific CD4 T cell responses detected following 9hr (left) or 18hr (right) stimulation. Data is shown as background subtracted in (A) and as fold-increase (stimulated / "UN") in (B). n = 3–5 independent, HIV-infected individuals.
(TIFF)

S2 Fig Overlap between antigen-specific populations. (A,B) Quantification of the frequency of antigen-specific CD4 T cells detected using 3 pairs of human AIM markers following a 9hr (A) or 18hr (B) stimulation in the presence (“+CD40b”, dark circles) or absence (“no CD40b”, light squares) of CD40 blocking antibody. Cells were stimulated with either HIV-1 Gag, HBV, SEB or left unstimulated. Blue indicates CD69^+^CD40L^+^, red indicates OX40^+^CD25^+^, and yellow indicates OX40^+^PD-L1^+^. n = 2 independent donors. (C) Quantification of CD69 expression on OX40^+^CD25^+^ CD4 T cells following 18 hr of antigen stimulation. Antigens are illustrated by different colors; HIV Gag (purple), HBV (green), hCMV (blue), HIV Env (orange). n = 3–5.
(TIFF)

S3 Fig Limited role of bystander activation in detection of antigen-specific cells by AIM assays. (A) A primary cell line from a HIV-infected individual was generated against the HIV Gag antigen. Approximately 30% of the cells were antigen-specific, as determined by intracellular cytokine staining for TNFα after 6 hr stimulation (B). The viability of CellVue-labeled PBMCs was assessed at all ratios of CD8-depleted cell line to PBMCs following an 18hr coculture and stimulation with CMV pp65 peptide pool. n = 2 independent donors and experiments. Error bars represent mean + SD.
(TIFF)

S4 Fig Treg cells in *M*. *mulatta* LN. (A) Quantification of Foxp3^+^Helios^+^ Treg cells within the AIM^+^ CD4 T cell population following BG505 stimulation for 18 hrs. n = 12 *M*. *mulatta* LN. (B) Example flow plot of Foxp3 and Helios expression in OX40^+^CD25^+^ *M*. *mulatta* LN CD4 T cells following BG505 antigen stimulation. (C) Comparison of the proportion of OX40^+^CD25^+^ nTreg cells (Foxp3^+^Helios^+^) within the total CD4 T cell population in *M*. *mulatta* LN following to BG505 stimulation. n = 8. (D) Example flow plot of CD39 and Foxp3 expression of total CD4 T cells in *M*. *mulatta* LN after 18 hours of incubation (no stimulation). (E) Venn diagram showing the overlap between Foxp3 and CD39 expression on total CD4 T cells in *M*. *mulatta* LN after 18 hours of incubation at 37°C (no stimulation). Numbers shown are mean; n = 8. (F) Example staining of 4-1BB in human PBMC following tetanus peptide pool stimulation, compared to alternative AIM marker combinations. (G) Quantification of signal detected in human PBMC following tetanus peptide pool stimulation; 4-1BB^+^OX40^+^ in purple, OX40^+^CD25^+^ in red, and PD-L1^+^OX40^+^ in yellow. n = 6 animals.
(TIFF)

S1 Table Antibody panel for the OX40/CD25 AIM assay. (TIFF)

S2 Table Antibody panel for the CD69/CD40L AIM assay. (TIFF)

S3 Table Antibody panel for the combined OX40/CD25 + CD69/CD40L AIM assay. (TIFF)

S4 Table Antibody panel for the combined ICS + OX40/CD25 AIM assay. (TIFF)

S5 Table Antibody panel for the OX40/CD25 AIM assay, used to investigate bystander activation in human PBMC in the transwell plate assay. (TIFF)

S6 Table Antibody panel used to quantify the antigen-specificity of CD4 T cell lines. (TIFF)

S7 Table Antibody panel used to investigate bystander activation in human PBMC, via the coculture of an antigen-specific CD4 T cell line and autologous PBMCs. (TIFF)

S8 Table Antibody panel used to quantify Treg cells and CD39 coexpression within the OX40/CD25/PD-L1 human AIM assay. (TIFF)

S9 Table Antibody panel used to quantify Treg cells within the combined OX40/CD25/PD-L1 + CD69/CD40L human AIM assay. (TIFF)

S10 Table Antibody panel used to quantify Treg cells and CD39 coexpression within the OX40/CD25 M. mulatta AIM assay. (TIFF)

S11 Table Antibody panel used to quantify Treg cells within the OX40/CD25/4-1BB M. mulatta AIM assay. (TIFF)
