# Supplementary figures and images for: Comparative analysis of activation induced marker (AIM) assays for sensitive identification of antigen-specific CD4 T cells
Source: PLoS One. 2017 Oct 24;12(10):e0186998. doi: 10.1371/journal.pone.0186998 (PMC5655442; doi:10.1371/journal.pone.0186998)

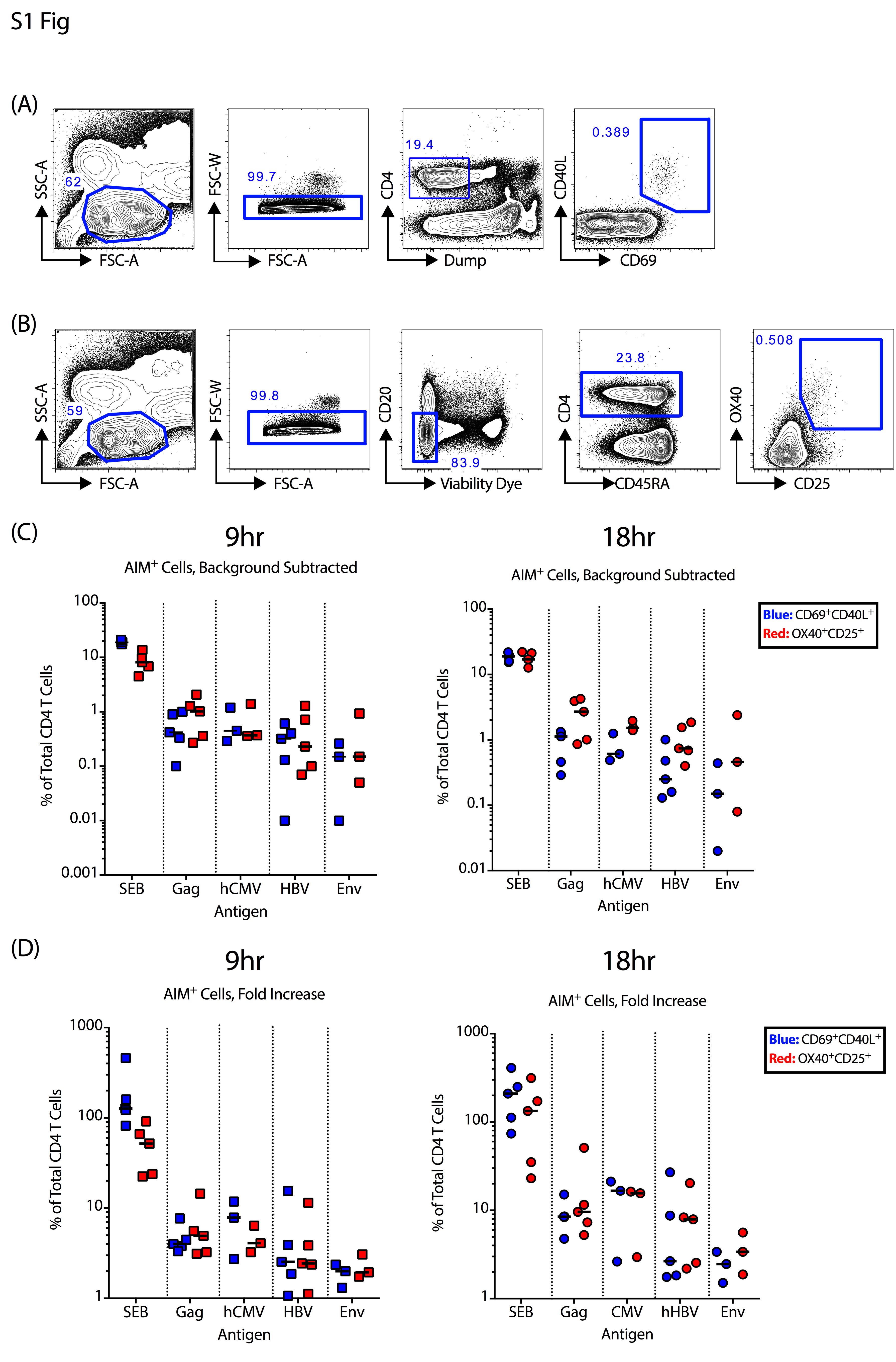

Supplement: S1 Fig — (A) Example flow plot gating strategy for the CD40L/CD69 Assay (dump channel contains a viability dye, CD8, CD14, CD16, and CD20) shown for HIV-Gag stimulated PBMCs. (B) Example flow plot gating strategy of the OX40/CD25 Assay shown for HIV-Gag stimulated PBMCs. (C,D) Quantification of antigen-specific CD4 T cell responses detected following 9hr (left) or 18hr (right) stimulation. Data is shown as background subtracted in (A) and as fold-increase (stimulated / "UN") in (B). n = 3–5 independent, HIV-infected individuals. (TIFF) [file pone.0186998.s001.tiff]

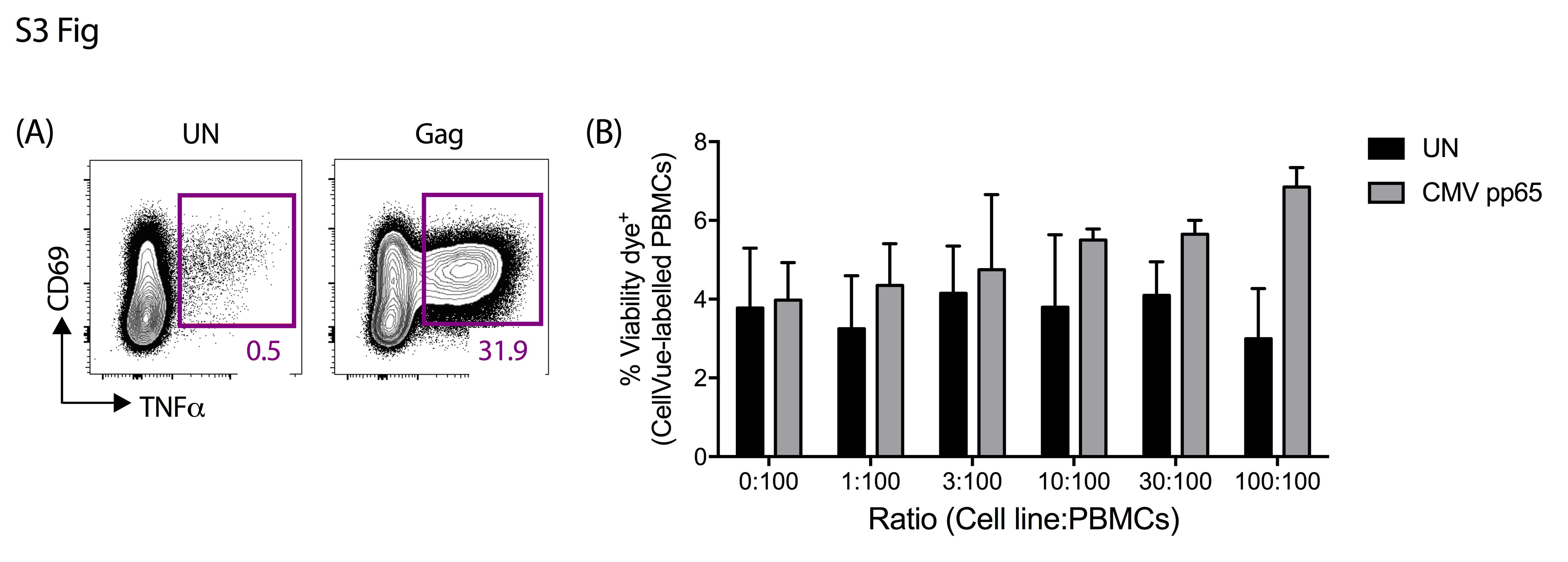

Supplement: S3 Fig — (A) A primary cell line from a HIV-infected individual was generated against the HIV Gag antigen. Approximately 30% of the cells were antigen-specific, as determined by intracellular cytokine staining for TNFα after 6 hr stimulation (B). The viability of CellVue-labeled PBMCs was assessed at all ratios of CD8-depleted cell line to PBMCs following an 18hr coculture and stimulation with CMV pp65 peptide pool. n = 2 independent donors and experiments. Error bars represent mean + SD. (TIFF) [file pone.0186998.s003.tiff]

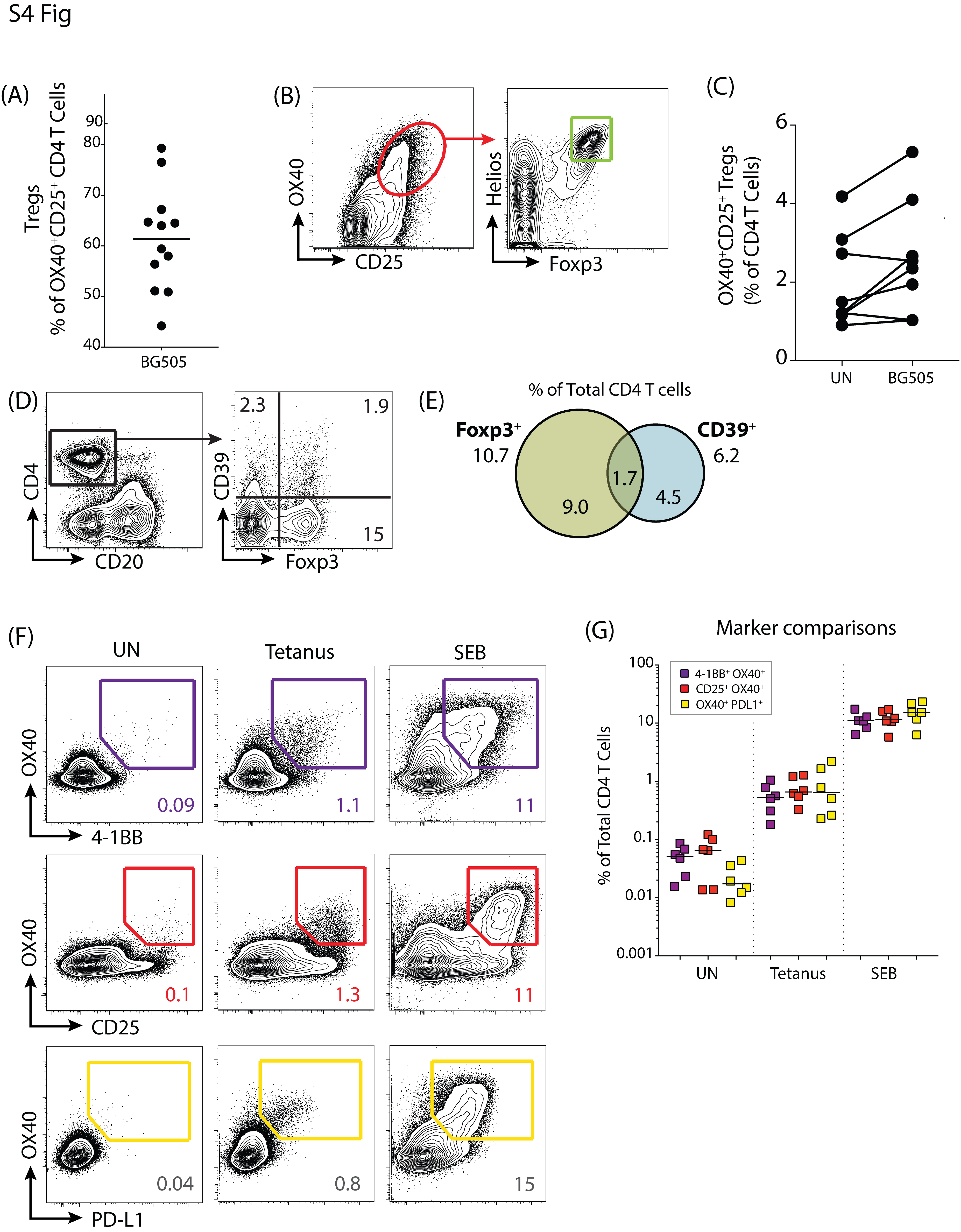

Supplement: S4 Fig — (A) Quantification of Foxp3+Helios+ Treg cells within the AIM+ CD4 T cell population following BG505 stimulation for 18 hrs. n = 12 M. mulatta LN. (B) Example flow plot of Foxp3 and Helios expression in OX40+CD25+ M. mulatta LN CD4 T cells following BG505 antigen stimulation. (C) Comparison of the proportion of OX40+CD25+ nTreg cells (Foxp3+Helios+) within the total CD4 T cell population in M. mulatta LN following to BG505 stimulation. n = 8. (D) Example flow plot of CD39 and Foxp3 expression of total CD4 T cells in M. mulatta LN after 18 hours of incubation (no stimulation). (E) Venn diagram showing the overlap between Foxp3 and CD39 expression on total CD4 T cells in M. mulatta LN after 18 hours of incubation at 37°C (no stimulation). Numbers shown are mean; n = 8. (F) Example staining of 4-1BB in human PBMC following tetanus peptide pool stimulation, compared to alternative AIM marker combinations. (G) Quantification of signal detected in human PBMC following tetanus peptide pool stimulation; 4-1BB+OX40+ in purple, OX40+CD25+ in red, and PD-L1+OX40+ in yellow. n = 6 animals. (TIFF) [file pone.0186998.s004.tiff]

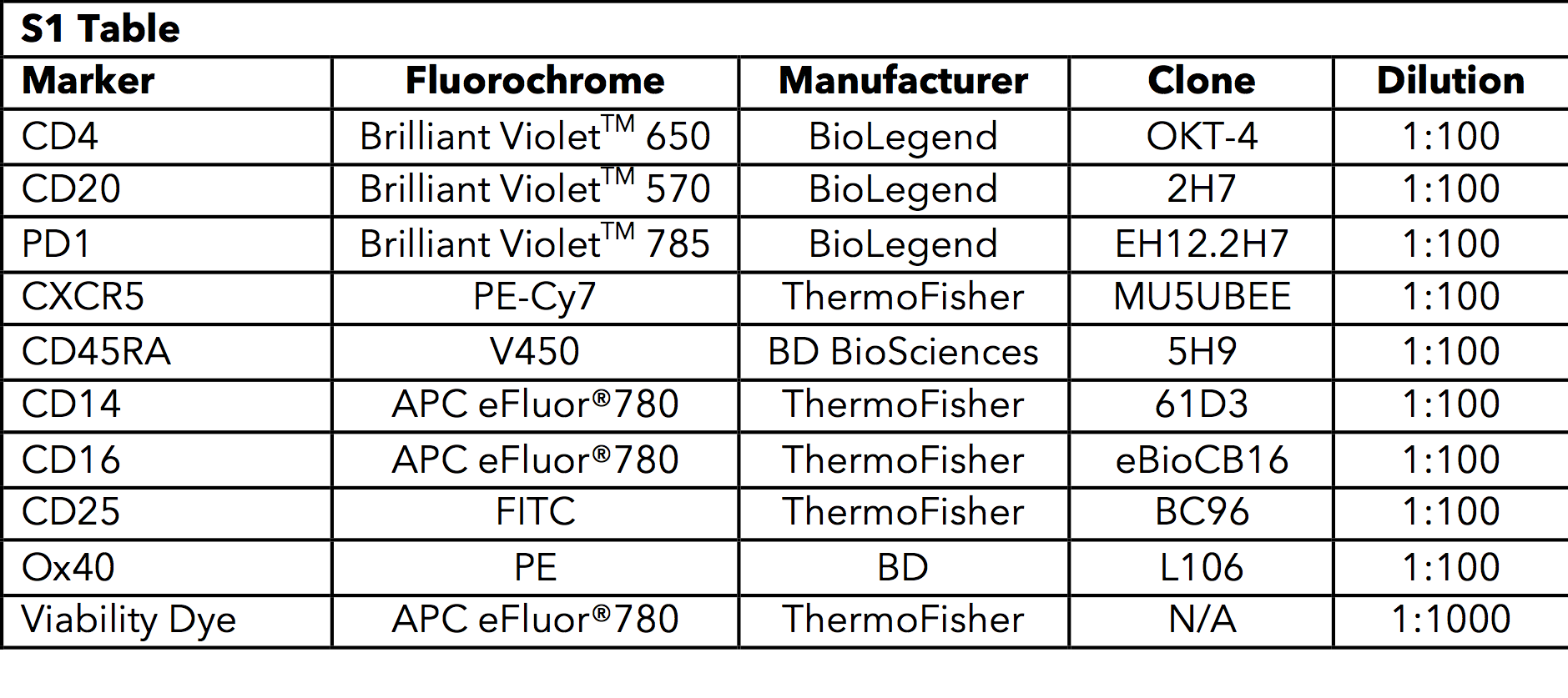

Supplement: S1 Table — (TIFF) [file pone.0186998.s005.tiff]

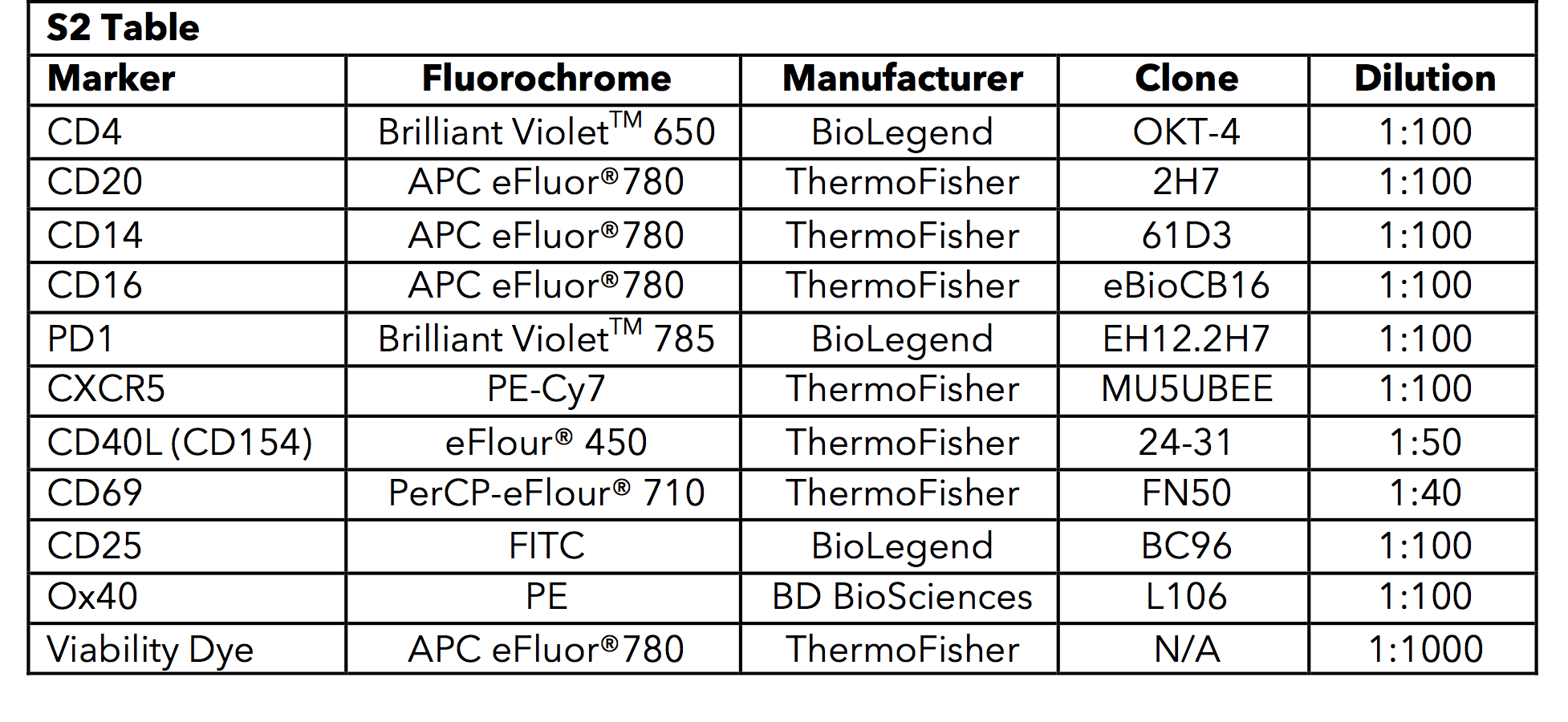

Supplement: S2 Table — (TIFF) [file pone.0186998.s006.tiff]

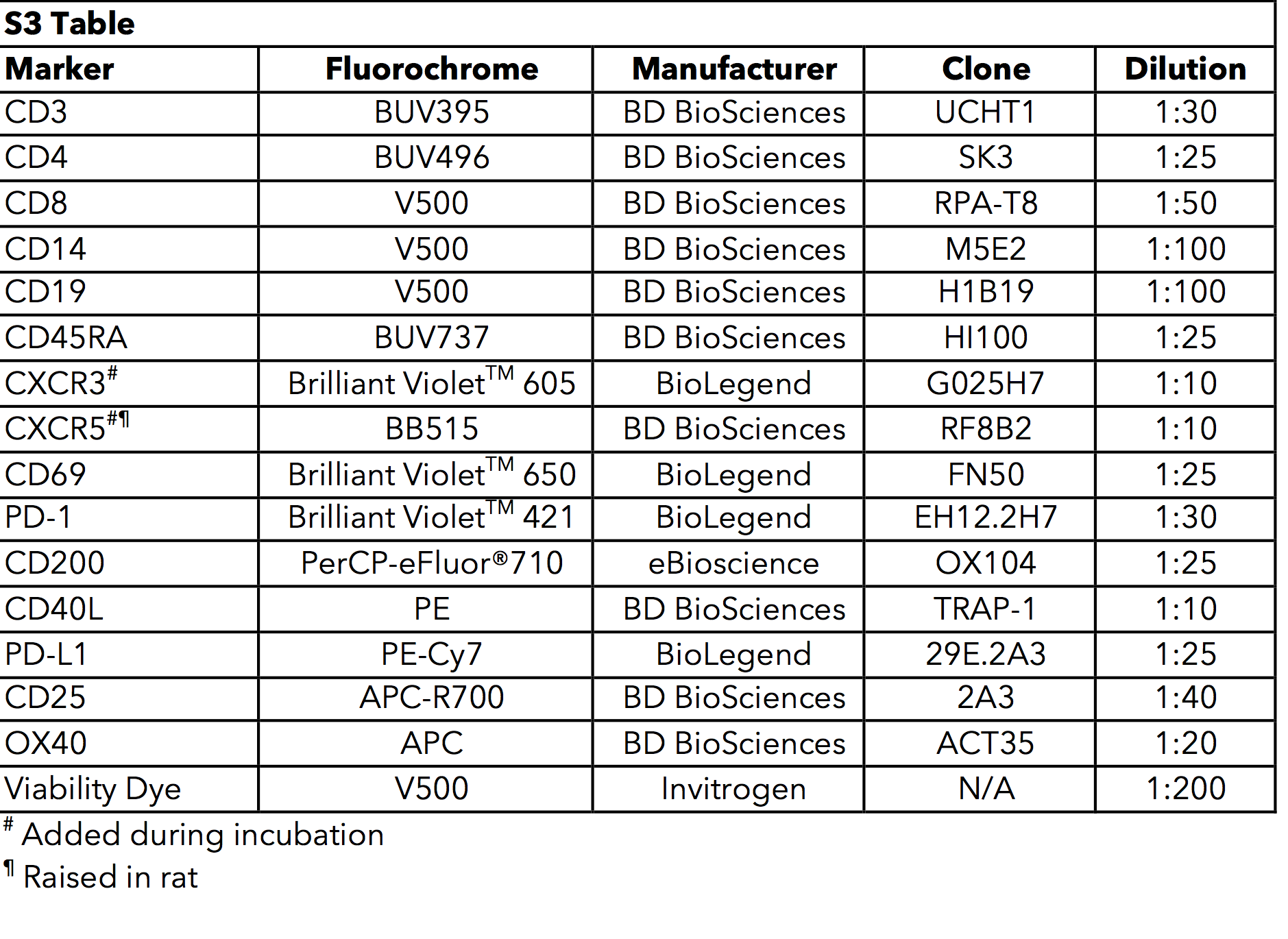

Supplement: S3 Table — (TIFF) [file pone.0186998.s007.tiff]

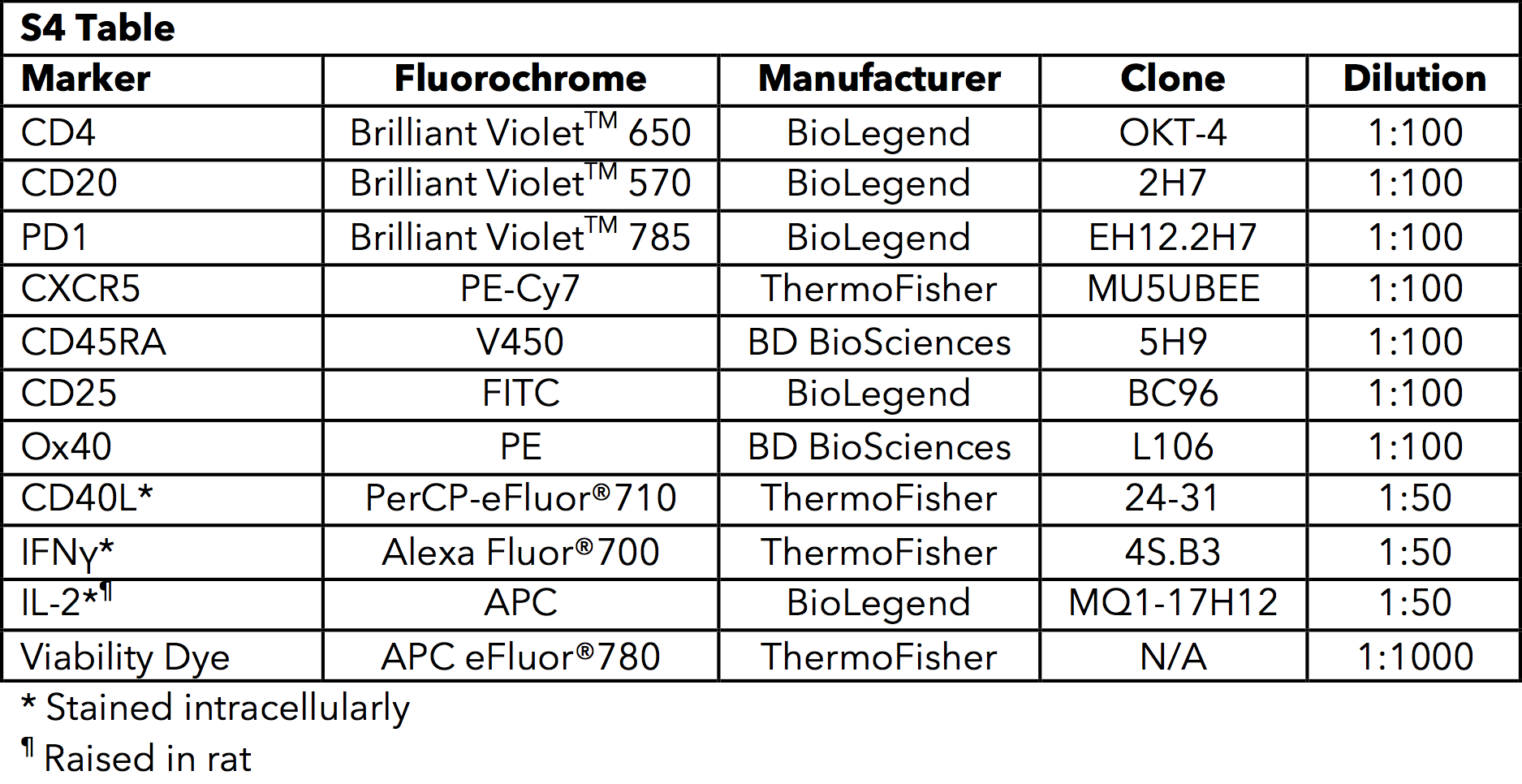

Supplement: S4 Table — (TIFF) [file pone.0186998.s008.tiff]

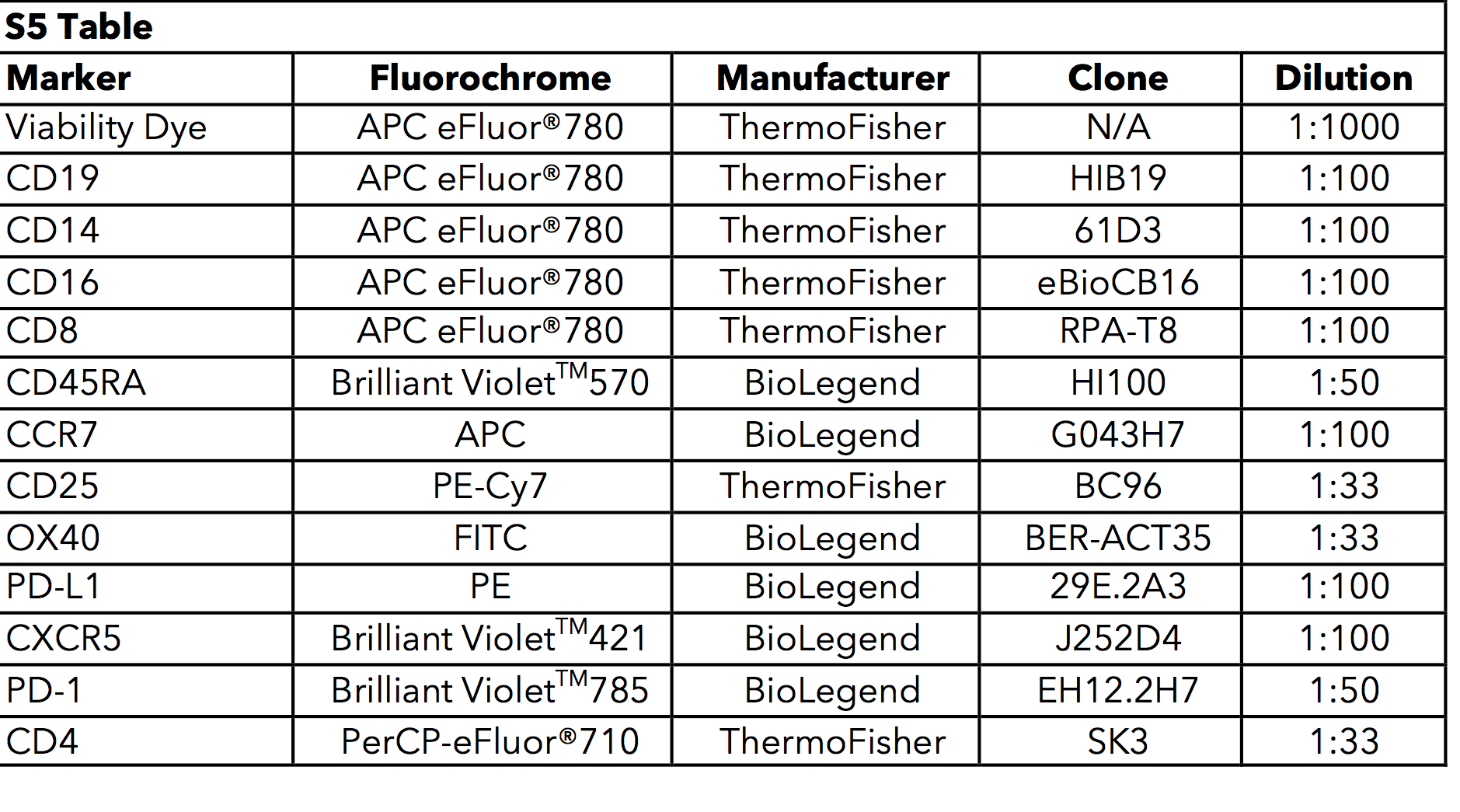

Supplement: S5 Table — (TIFF) [file pone.0186998.s009.tiff]

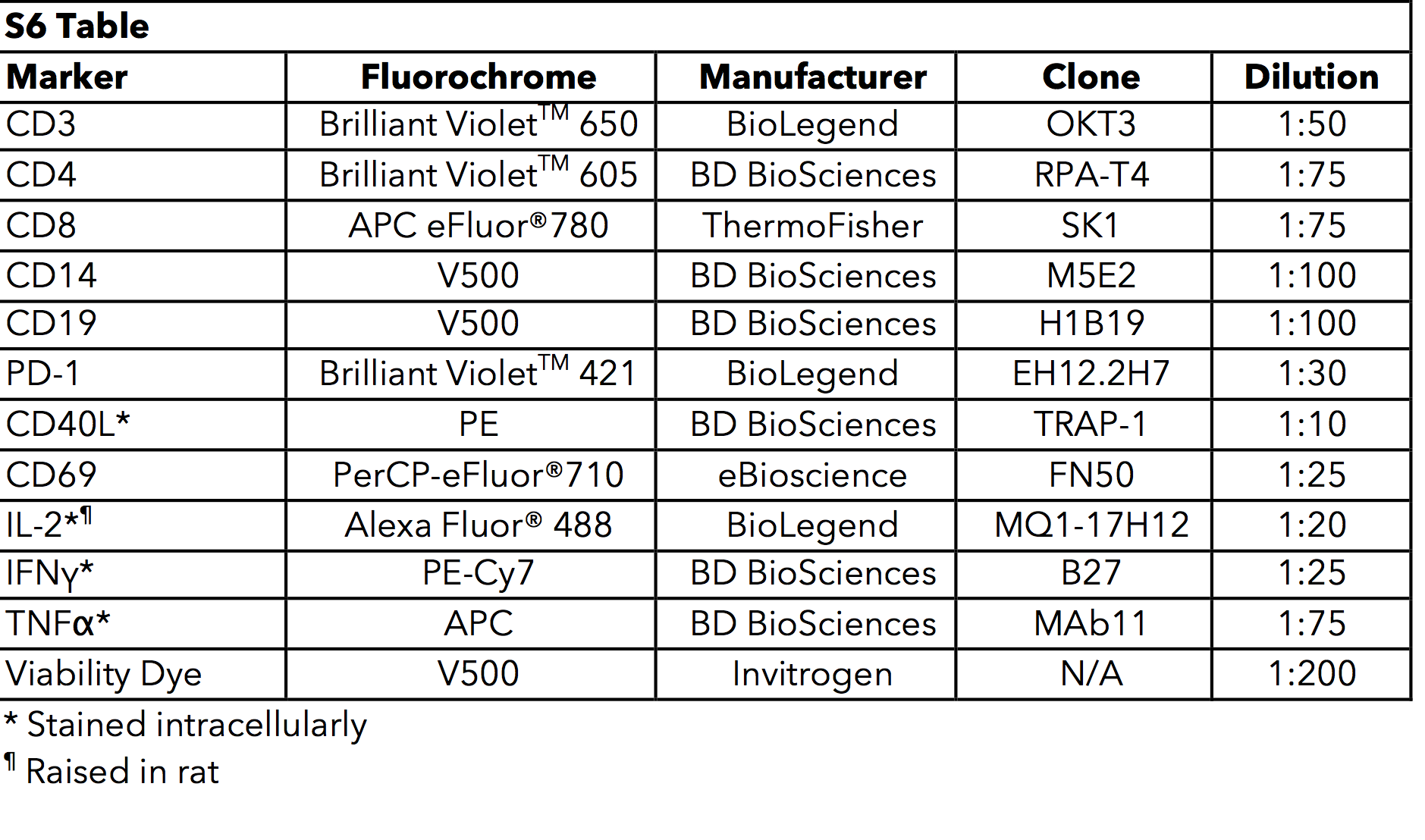

Supplement: S6 Table — (TIFF) [file pone.0186998.s010.tiff]

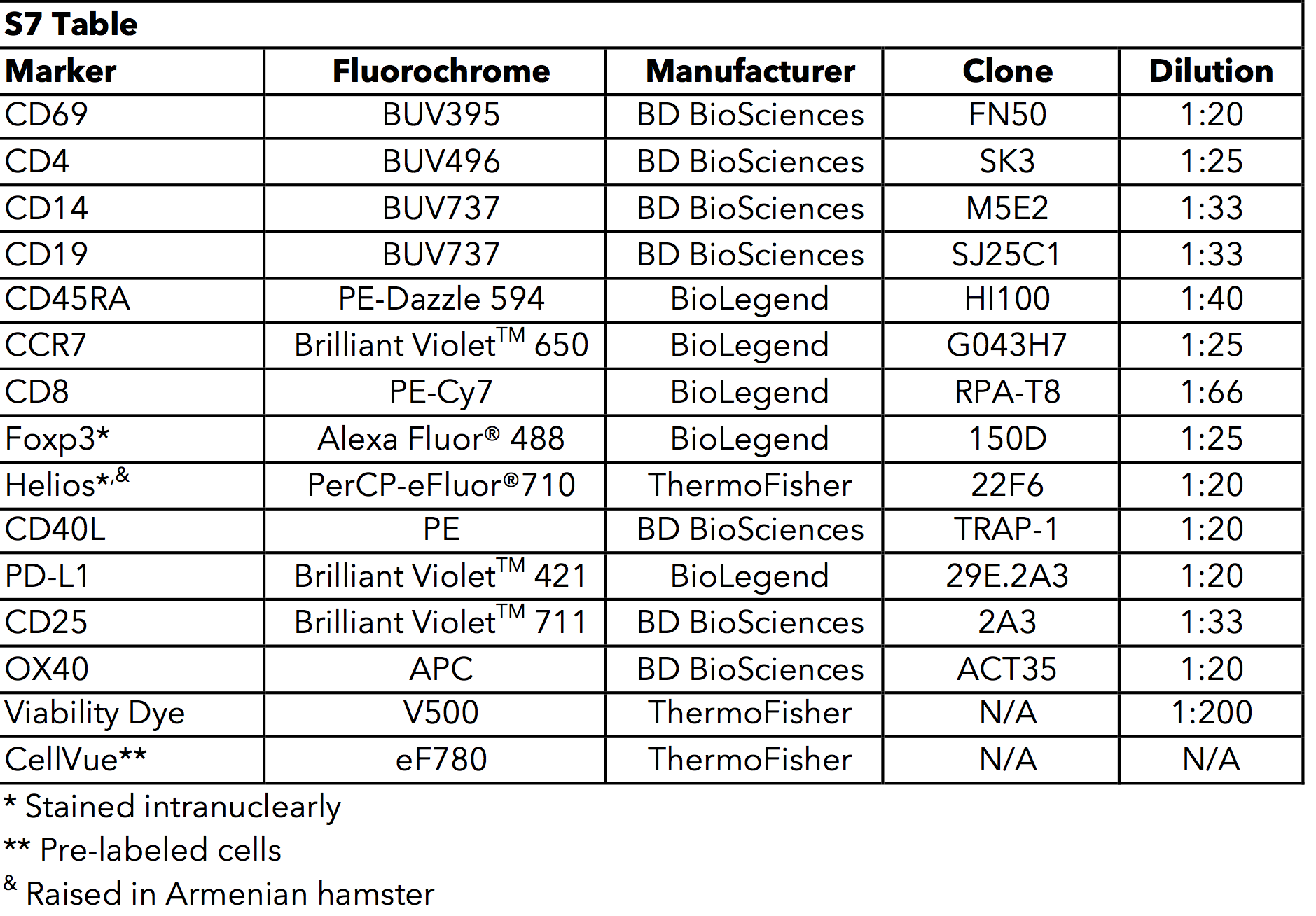

Supplement: S7 Table — (TIFF) [file pone.0186998.s011.tiff]

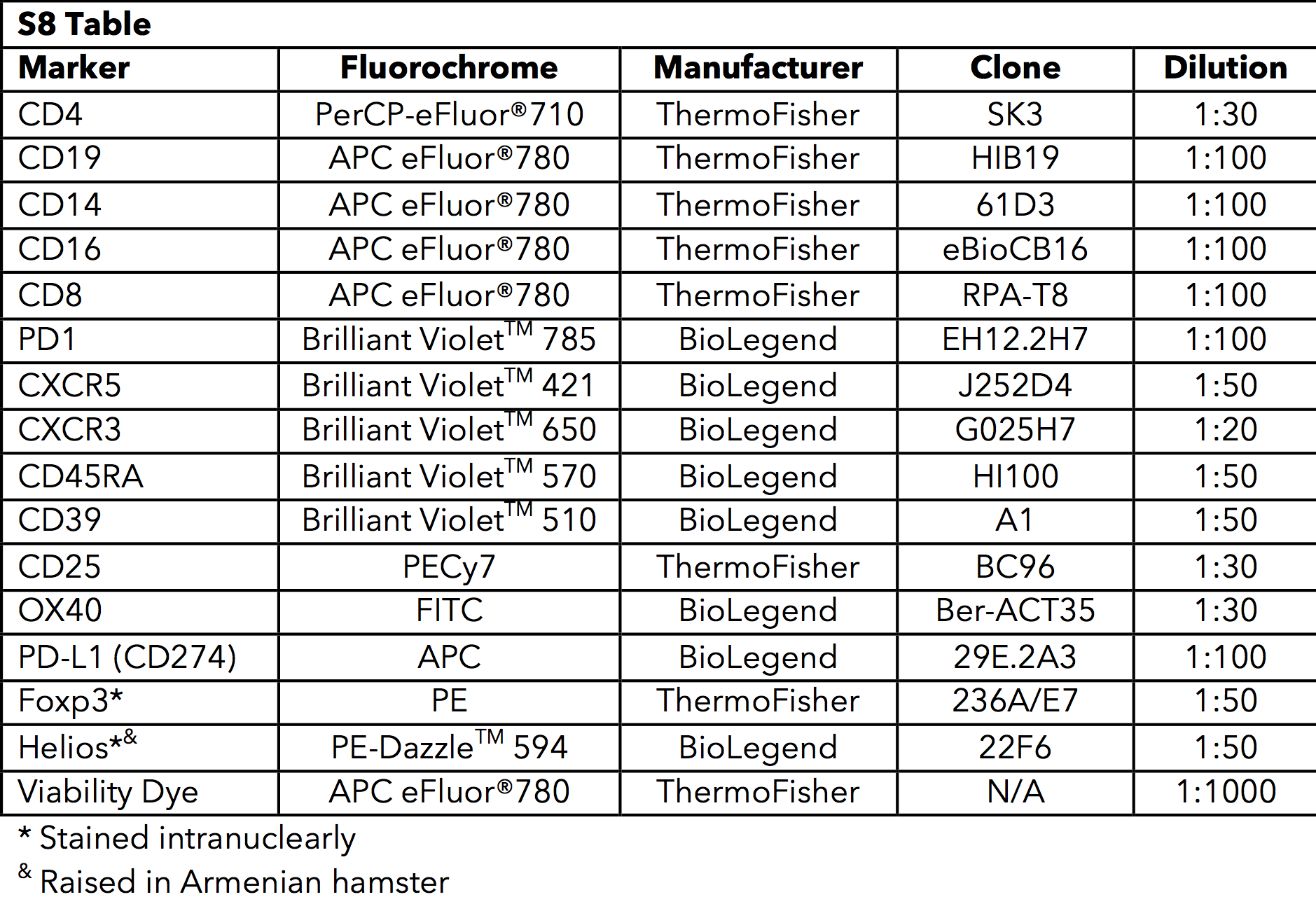

Supplement: S8 Table — (TIFF) [file pone.0186998.s012.tiff]

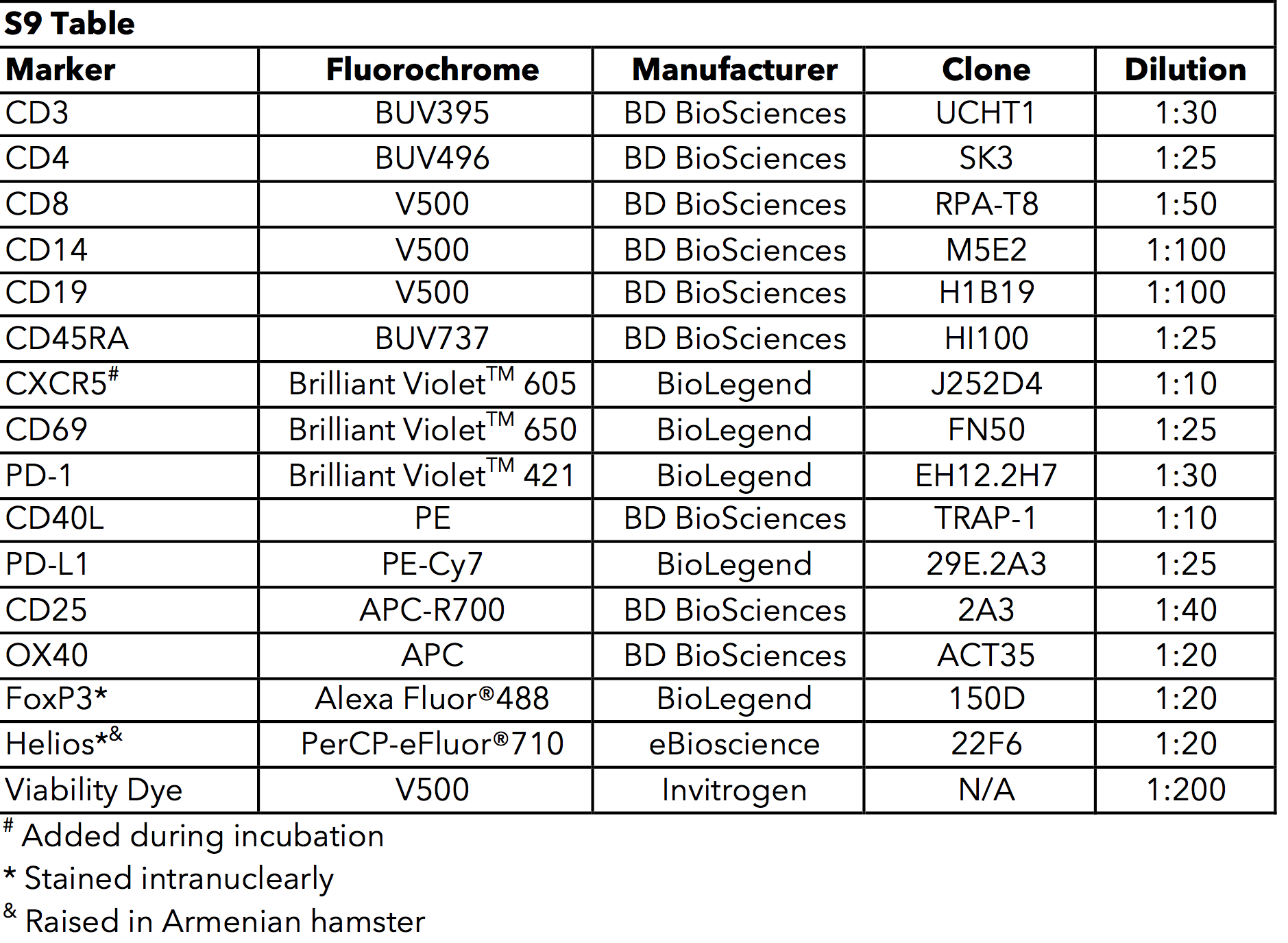

Supplement: S9 Table — (TIFF) [file pone.0186998.s013.tiff]

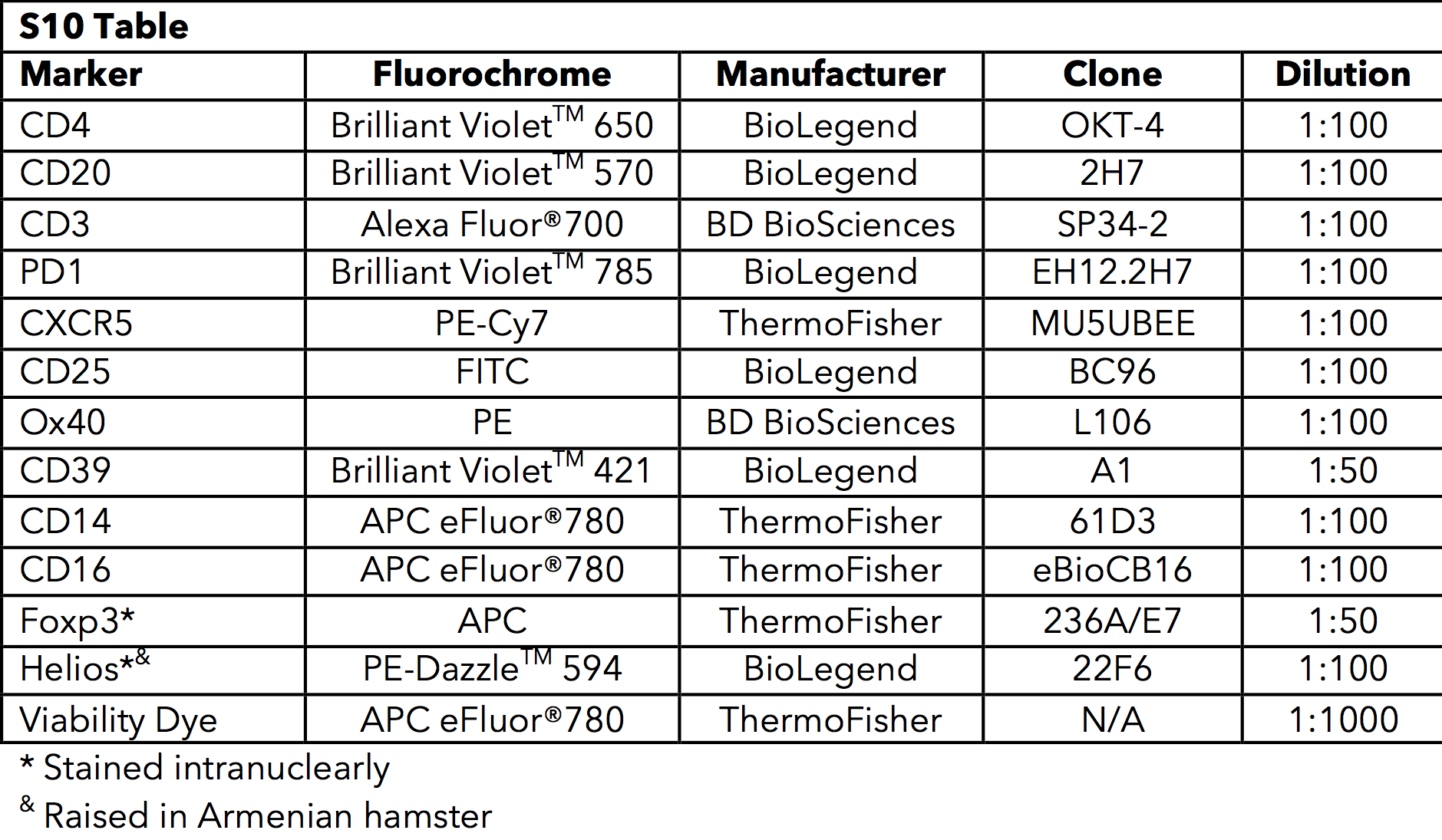

Supplement: S10 Table — (TIFF) [file pone.0186998.s014.tiff]

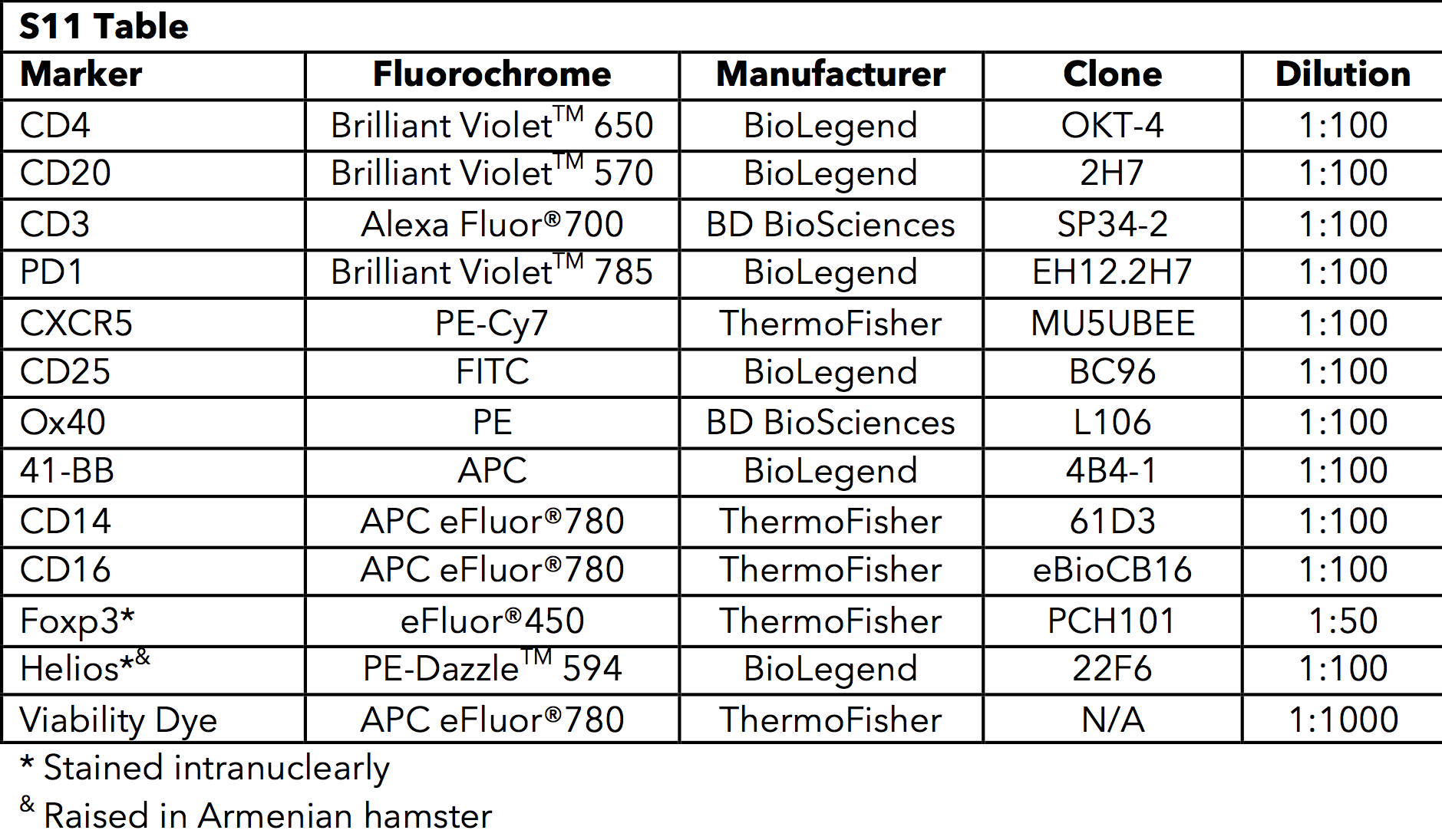

Supplement: S11 Table — (TIFF) [file pone.0186998.s015.tiff]
